# RIPK1 and Caspase-8 Ensure Chromosome Stability Independently of Their Role in Cell Death and Inflammation

**DOI:** 10.1016/j.molcel.2018.11.010

**Published:** 2019-02-07

**Authors:** Gianmaria Liccardi, Laura Ramos Garcia, Tencho Tenev, Alessandro Annibaldi, Arnaud J. Legrand, David Robertson, Rebecca Feltham, Holly Anderton, Maurice Darding, Nieves Peltzer, Marius Dannappel, Hannah Schünke, Luca L. Fava, Manuel D. Haschka, Timo Glatter, Alexey Nesvizhskii, Alexander Schmidt, Philip A. Harris, John Bertin, Peter J. Gough, Andreas Villunger, John Silke, Manolis Pasparakis, Katiuscia Bianchi, Pascal Meier

**Affiliations:** 1 The Breast Cancer Now Toby Robins Research Centre, Institute of Cancer Research, Mary-Jean Mitchell Green Building, Chester Beatty Laboratories, 237 Fulham Road, London SW3 6JB, UK; 2 The Walter and Eliza Hall Institute, 1G Royal Parade, Parkville, Victoria 3052, Australia; 3 Centre for Cell Death, Cancer, and Inflammation (CCCI), UCL Cancer Institute, University College, London WC1E 6BT, UK; 4 Institute for Genetics, Centre for Molecular Medicine (CMMC) and Cologne Excellence Cluster on Cellular Stress Responses in Aging-Associated Diseases (CECAD), University of Cologne, 50931 Cologne, Germany; 5 Division of Dev. Immunology, Biocenter, Medical University of Innsbruck, Innsbruck, A-6020, Austria; 6 Proteomics Core Facility, Biocentrum of the University of Basel, Basel, Switzerland; 7 Max Planck Institute for Terrestrial Microbiology, Karl-von-Frisch Str. 10, 35043 Marburg, Germany; 8 Department of Pathology, Department of Computational Medicine & Bioinformatics, University of Michigan, Ann Arbor, MI, USA; 9 Pattern Recognition Receptor Discovery Performance Unit, Immuno-Inflammation Therapeutic Area, GlaxoSmithKline, Collegeville, PA 19426, USA; 10 Tyrolean Cancer Research Institute, A-6020 Innsbruck, Austria; 11 Barts Cancer Institute, Queen Mary, John Vane Science Centre, University of London, Charterhouse Square, London EC1M 6BQ, UK

**Keywords:** RIPK1, caspase-8, cell death, cell cycle, mitosis, PLK1, BUBR1, chromosomal instability, ripoptosome, cancer

## Abstract

Receptor-interacting protein kinase (RIPK) 1 functions as a key mediator of tissue homeostasis via formation of Caspase-8 activating ripoptosome complexes, positively and negatively regulating apoptosis, necroptosis, and inflammation. Here, we report an unanticipated cell-death- and inflammation-independent function of RIPK1 and Caspase-8, promoting faithful chromosome alignment in mitosis and thereby ensuring genome stability. We find that ripoptosome complexes progressively form as cells enter mitosis, peaking at metaphase and disassembling as cells exit mitosis. Genetic deletion and mitosis-specific inhibition of *Ripk1* or *Caspase-8* results in chromosome alignment defects independently of MLKL. We found that Polo-like kinase 1 (PLK1) is recruited into mitotic ripoptosomes, where PLK1’s activity is controlled via RIPK1-dependent recruitment and Caspase-8-mediated cleavage. A fine balance of ripoptosome assembly is required as deregulated ripoptosome activity modulates PLK1-dependent phosphorylation of downstream effectors, such as BUBR1. Our data suggest that ripoptosome-mediated regulation of PLK1 contributes to faithful chromosome segregation during mitosis.

## Highlights

• A RIPK1/FADD/Caspase-8/cFLIP complex (ripoptosome) forms during mitosis
• The mitotic kinase PLK1 binds to RIPK1 and is recruited into mitotic ripoptosomes
• PLK1 is cleaved by Caspase-8 in a RIPK1-dependent manner
• Deletion and inhibition of RIPK1 or Caspase-8 lead to chromosomal instability

## Graphical Abstract

**Figure:**
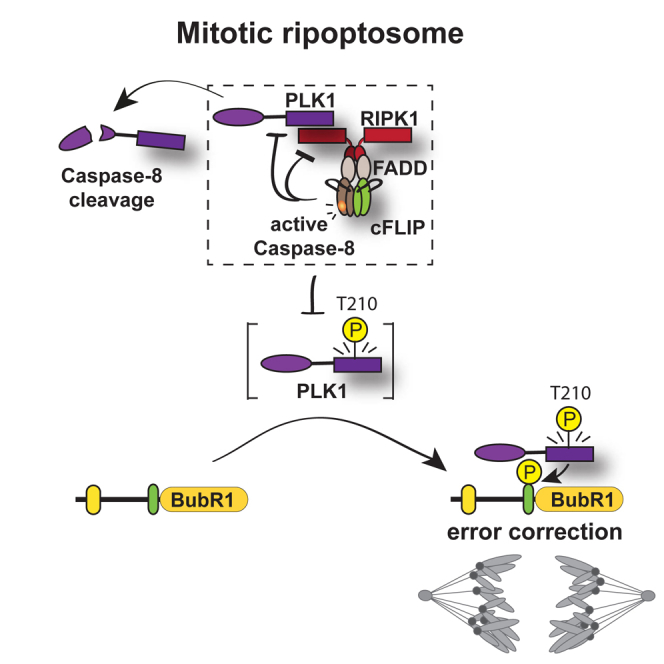

## Teaser

Liccardi et al. show that ripoptosome complexes assemble during physiological mitosis and regulate PLK1 activity via RIPK1-dependent recruitment and Caspase-8-mediated cleavage. These results reveal a direct role for RIPK1 and Caspase-8 in controlling chromosome segregation during mitosis.

## Introduction

RIPK1 functions as a critical signaling node in various innate immune pathways (Rickard et al., 2014, Takahashi et al., 2014, Weinlich and Green, 2014). Its activity is subject to tight regulation, most notably by phosphorylation, ubiquitylation, and Caspase-8 (Casp8)-mediated cleavage. Proper regulation of the kinase activity and scaffolding function of RIPK1 is crucial, as deregulation of RIPK1 is associated with various human pathologies (Silke et al., 2015).

Following its activation, RIPK1 associates with the endopeptidase Casp8 and cellular FLICE-like inhibitor protein (cFLIP_L_) through the adaptor molecule Fas-associated protein with Death Domain (FADD) to form a RIPK1-based Casp8 activating complex, variably referred to as complex-IIB (Petersen et al., 2007, Wang et al., 2008), ripoptosome (Tenev et al., 2011), or necrosome when the RIP kinase family member RIPK3 is also recruited to this complex (Pasparakis and Vandenabeele, 2015). For simplicity, we will subsequently refer to this RIPK1/FADD/Casp8/cFLIP multiprotein complex as “ripoptosome.” Formation of the ripoptosome frequently occurs under physiological conditions without killing the cell, generating a burst of sub-lethal caspase activity (Boatright et al., 2004, Micheau et al., 2002) that causes cleavage of RIPK1 and RIPK3 and destabilization of ripoptosome complexes (Feng et al., 2007, Oberst et al., 2011). IAP-mediated ubiquitylation of RIPK1 and Casp8 represents a further control point that restricts the lethal potential of RIPK1 and the ripoptosome (Tenev et al., 2011).

Beyond its role in regulating cell death in some cell types, Casp8 also fulfils non-cell-death functions (Maelfait and Beyaert, 2008). For example, evidence exists that indicates a possible tumor suppressor function of Casp8. Accordingly, deficiency of *Casp8* facilitates cellular transformation *in vitro* (Krelin et al., 2008), acts as driver mutation in breast cancer (Stephens et al., 2012) and B cell lymphoma (Hakem et al., 2012), and is frequently found to be mutated in hepatocellular carcinomas (Soung et al., 2005b) and advanced gastric cancer (Soung et al., 2005a). Further, loss of *Casp8* expression is associated with human neuroblastomas with N-Myc amplification (Teitz et al., 2000), small-cell lung carcinoma (Hopkins-Donaldson et al., 2003), and relapsed glioblastoma multiforme (Martinez et al., 2007). Moreover, Casp8 reportedly is essential for maintaining chromosomal stability (Hakem et al., 2012), independent of its role in cell death. Despite these data, compelling evidence is lacking to support a direct causal role for *Casp8* inactivation in the generation of cancer chromosomal instability.

By studying why Casp8 is essential for maintaining chromosomal stability, we identified RIPK1 and Casp8 (ripoptosome complexes) as negative regulators of polo-like kinase 1 (PLK1), a key kinase that regulates chromosomal segregation, spindle assembly checkpoint, and maintenance of genomic integrity (Medema et al., 2011, Zitouni et al., 2014). We noticed that ripoptosome complexes form physiologically during mitosis and that active PLK1 is recruited into these complexes by RIPK1. Upon its recruitment, PLK1 is cleaved at D457 by Casp8, similarly to other ripoptosome components. In the absence of *Ripk1*, active PLK1 accumulates, resulting in excessive phosphorylation of BUBR1 and mitotic defects. Conversely, enhancing ripoptosome formation during mitosis suppresses the interaction of PLK1 with its substrate BUBR1, resulting in hypo-phosphorylation of BUBR1 and defects in chromosome congression and segregation. Collectively, our data demonstrate that deregulation of key ripoptosome components causes chromosomal instability, independently of their role in cell death and inflammation. Our data provide a likely explanation as to why loss of Casp8 and low levels of *Ripk1* can be driver mutations in certain types of cancer, leading to chromosome instability that may favor tumor evolution, heterogeneity, acquisition of drug resistance, and heightened risk for tumor relapse.

## Results

### The Ripoptosome Assembles during Physiological Mitosis

**Figure 1:**
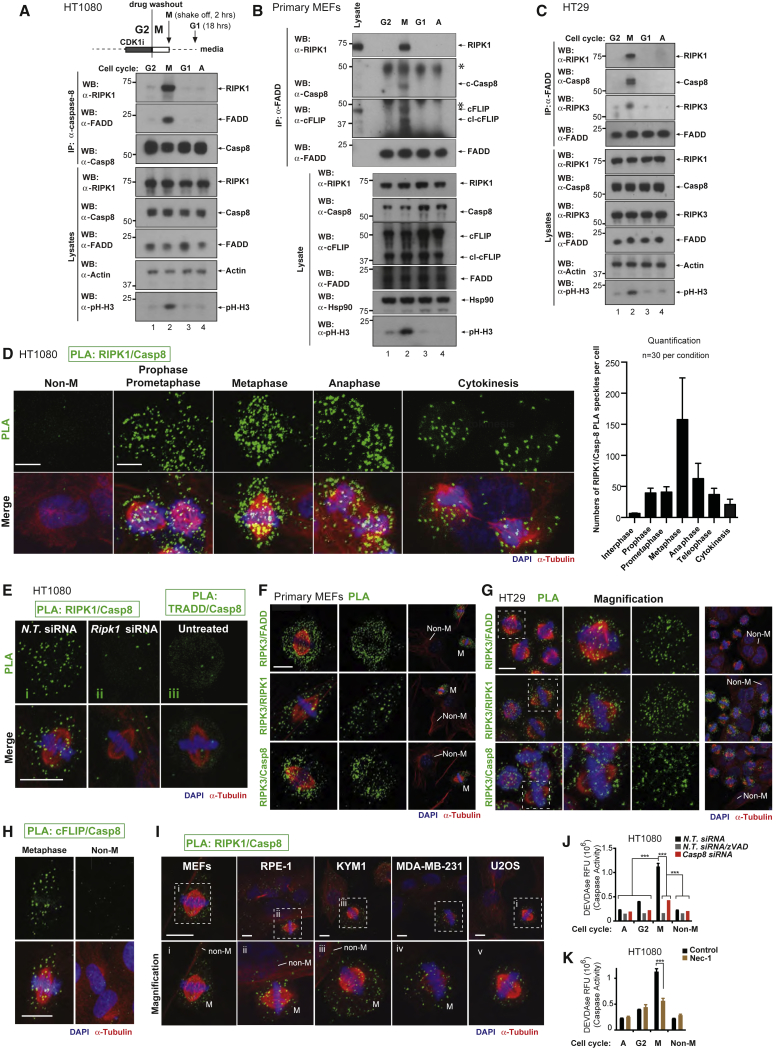
The Ripoptosome Forms During Normal Mitosis (A–C) Human HT1080 (A), MEFs (B), and HT29 (C) cells were synchronized, and lysates from asynchronous or synchronized cells were immunoprecipitated with anti-Casp8 (HT1080) or anti-FADD (MEFs, HT29) antibodies. Immunoblot analysis using the indicated antibodies is shown. The synchronization scheme and collection points are indicated above. (D) *In situ* PLA detection of RIPK1 and Casp8 in HT1080 cells. Green dots indicate PLA signals of RIPK1/Casp8 complexes. The panel on the right shows quantifications of RIPK1/Casp8 PLA speckles (mean ± SD from three independent experiments). In each experiment, 10 cells were counted for each mitotic stage. Scale bars: 10 μm. (E–G) *In situ* PLA detection using antibodies against the indicated proteins. Green dots indicate proximity signals of RIPK1/Casp8 or TRADD/Casp8 in HT1080 (E), RIPK3/FADD, RIPK3/RIPK1, or RIPK3/Casp8 in MEFs (F) or HT29 (G). Scale bars: 10 μm. (H) *In situ* PLA detection of cFLIP and Casp8 in HT1080 cells. Green dots indicate proximity signals between cFLIP and Casp8. Scale bars: 10 μm. (I) *In situ* PLA detection of RIPK1 and Casp8 in the indicated cell lines. Green dots indicate PLA signals of RIPK1/Casp8 complexes. Scale bars: 10 μm. (J and K) DEVDase caspase activity analysis using CDK1i-synchronized and released HT1080 cells treated with the indicated conditions.

Immunoprecipitation of Casp8 from cells in different stages of the cell cycle revealed that RIPK1, FADD, Casp8, and cFLIP associated during mitosis of HT1080, primary MEFs, and HT29 cells, suggesting that the ripoptosome can form during mitosis (Figures 1A–1C and S1A). To visualize ripoptosome complexes in their native state in intact cells, we utilized *in situ* proximity ligation assay (PLA) to detect RIPK1/Casp8 complexes (Orme et al., 2016). While ripoptosome formation was undetectable in G2, ripoptosome complexes steadily formed as cells entered mitosis (prophase), peaking at metaphase and declining as cells exited M-phase (Figure 1D). Although TRADD can also activate Casp8 (Anderton et al., 2018, Wang et al., 2008), we found no evidence for TRADD/Casp8 complexes during mitosis (Figure 1E). Additional PLA controls are provided in Figures S1B and S1C.

Ripoptosome complexes also formed in M-phases of unperturbed, non-synchronized cells (Figure S1D), indicating that this complex transiently forms during normal mitosis. PLA analysis detected mitosis-specific association of RIPK3/FADD, RIPK3/RIPK1, and RIPK3/Casp8 in MEFs (Figure 1F) and HT29 cells (Figure 1G) and confirmed cFLIP/Casp8 interactions in mitotic cells (Figures 1H and S1I). All antibodies were tested for PLA signal specificity (Figures S1E–S1I). Ripoptosome formation during mitosis also occurred in a panel of additional cell types, implying that ripoptosome formation during mitosis is a general phenomenon (Figure 1I). Using 3D reconstruction, RIPK1/Casp8 complexes appeared to be distributed throughout the nucleoplasm and not associated with the spindle, centrosomes, or kinetochores of mitotic cells (Figure S1J), which was in agreement with RIPK1- and Casp8-staining during mitosis (Figure S1K).

Consistent with the formation of an active ripoptosome in mitosis, cells in M-phase showed significantly higher levels of caspase activity than cells in other cell cycle phases (Figures 1J and S1L). Mitotic caspase activity was Casp8- and RIPK1-dependent as RNAi-mediated depletion of *Casp8*, or treatment with the RIPK1 kinase inhibitor Nec-1, suppressed this caspase activity (Figures 1J and 1K). Similarly, expression of the viral Casp8 inhibitor CrmA, or treatment with the caspase inhibitor zVAD-FMK, also suppressed caspase activity in M-phase (Figures 1J and S1L). Despite measurable levels of caspase activity, mitotic cells remained fully viable (Figure S1M, and data not shown). Consistently, mitotic cells were “unprimed” to die by a mitochondria-dependent form of apoptosis (Figure S1N). Further, RNAi-mediated depletion of *cFLIP* suppressed the caspase activity in M-phase, indicating the non-killing activity of this complex (Figure S1O). RNAi-mediated depletion of *Tnfr1*, *Trail-r1/r2*, and *CD95/Fas* did not prevent formation of Casp8/RIPK1 complexes (Figure S1P). Under the same condition, RNAi-mediated depletion of these death receptors suppressed cell death mediated by their respective ligands (Figure S1Q). Together, these data indicate that RIPK1/FADD/Casp8/cFLIP form a complex during mitosis (mitotic ripoptosome), generating a pulse of sub-lethal caspase-8 activity that is dependent on cFLIP and RIPK1 kinase activity.

### PLK1 Is Recruited to RIPK1 and the Ripoptosome

To gain further insights into the mitotic ripoptosome, we undertook a proteomic-based approach using RIPK1 as affinity reagent. In addition to identifying known interactors of RIPK1, the mass spectrometry experiment also identified PLK1 as a putative binding partner of RIPK1 (Figure S2A). Under the same conditions, PLK1 was not co-purified with GFP, LacZ, FADD, Casp8, and cFLIP (data not shown), implying that the identification of PLK1 is not due to nonspecific interaction.

**Figure 2:**
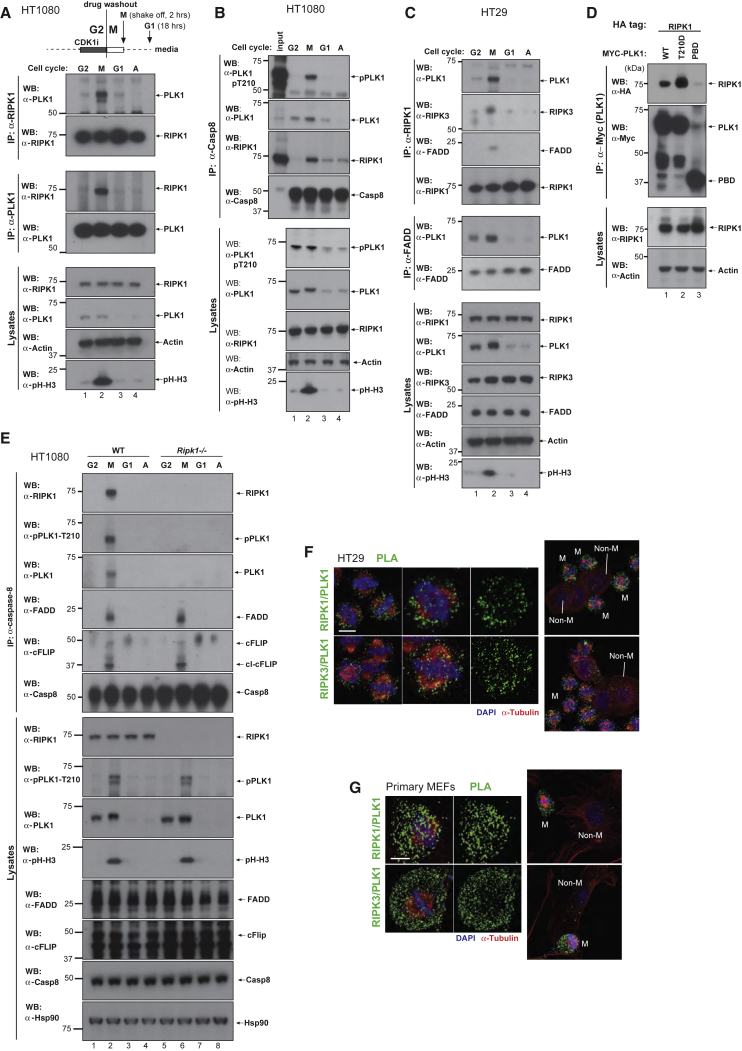
RIPK1 Interacts with PLK1 (A) HT1080 cells were synchronized with CDK1i and released. Lysates from asynchronous or synchronized HT1080 cells were immunoprecipitated with anti-RIPK1 or anti-PLK1 antibodies. Immunoblot analysis using the indicated antibodies is shown. (B) HT1080 cells were synchronized with CDK1i and released. Lysates from asynchronous or synchronized HT1080 cells were immunoprecipitated with anti-Casp8 antibody. Immunoblot analysis using the indicated antibodies is shown. (C) Lysates from synchronized and released HT29 cells were immunoprecipitated with anti-RIPK1 or anti-FADD antibodies. Immunoblot analysis using the indicated antibodies is shown. (D) The indicated constructs were co-expressed in HEK293T cells. Myc-immunoprecipitation was performed and RIPK1 interaction was assessed via western blot. (E) HT1080 cells were synchronized with CDK1i and released. Lysates from asynchronous or synchronized HT1080 cells were immunoprecipitated with anti-Casp8 antibody. Immunoblot analysis using the indicated antibodies is shown. (F) *In situ* PLA detection of PLK1/RIPK1 or PLK1/RIPK3 in CDK1i-synchronized and released HT29. Green dots indicate PLA speckles. Scale bars: 10 μm (G) *In situ* PLA detection of PLK1/RIPK1 or PLK1/RIPK3 in CDK1i-synchronized and released MEFs. Green dots indicate PLA speckles. Scale bars: 10 μm

Reciprocal immunoprecipitation of endogenous RIPK1 and PLK1 from cells in different stages of the cell cycle revealed that PLK1 and RIPK1 associated during mitosis (Figure 2A). Immunoprecipitation of endogenous Casp8, RIPK1, and FADD from mitotic cells also co-purified PLK1 along with other components of the ripoptosome (Figures 2B and 2C). No such interaction with PLK1 was observed outside of mitosis. While wild-type and the constitutively active form of PLK1 (PLK1-T210D) bound to RIPK1, the PBD domain of PLK1 in isolation failed to bind to RIPK1, suggesting that PLK1 binds to RIPK1 independently of PBD-mediated phospho-recognition (Figure 2D). Recruitment of PLK1 to ripoptosome complexes was strictly RIPK1-dependent, as PLK1 failed to be co-purified by Casp8 in the absence of RIPK1 (Figure 2E). Interestingly, both FADD and cFLIP associated with Casp8 independently of RIPK1, suggesting that RIPK1 does not nucleate or trigger this complex during mitosis. RIPK1 mutants, lacking the RHIM or DD domain, readily associated with PLK1 (Figure S2B), indicating that RIPK1 can bind to PLK1 independently of FADD, caspase-8, or RIPK3.

The interaction between RIPK1 and PLK1 in mitosis was also detectable using PLA with antibodies against RIPK1 and total PLK1 or phospho-specific PLK1 (anti-PLK1-pT210) (Figures S2C–S2F). Mitosis-specific interaction between RIPK1 and PLK1, as well as RIPK3 and PLK1, was also detected in HT29 and primary MEFs (Figures 2F and 2G), expanding this observation to additional cell types. RNAi-mediated depletion of *Tnfr1*, *Trail-r1/r2*, and *CD95/Fas* did not interfere with the formation of PLK1/RIPK1 complexes (Figure S2G). Together, our data demonstrate that PLK1, RIPK1, RIPK3, FADD, cFLIP_L_, and Casp8 form ripoptosome complexes in mitosis.

### The Ripoptosome Restrains PLK1 in Mitosis

**Figure 3:**
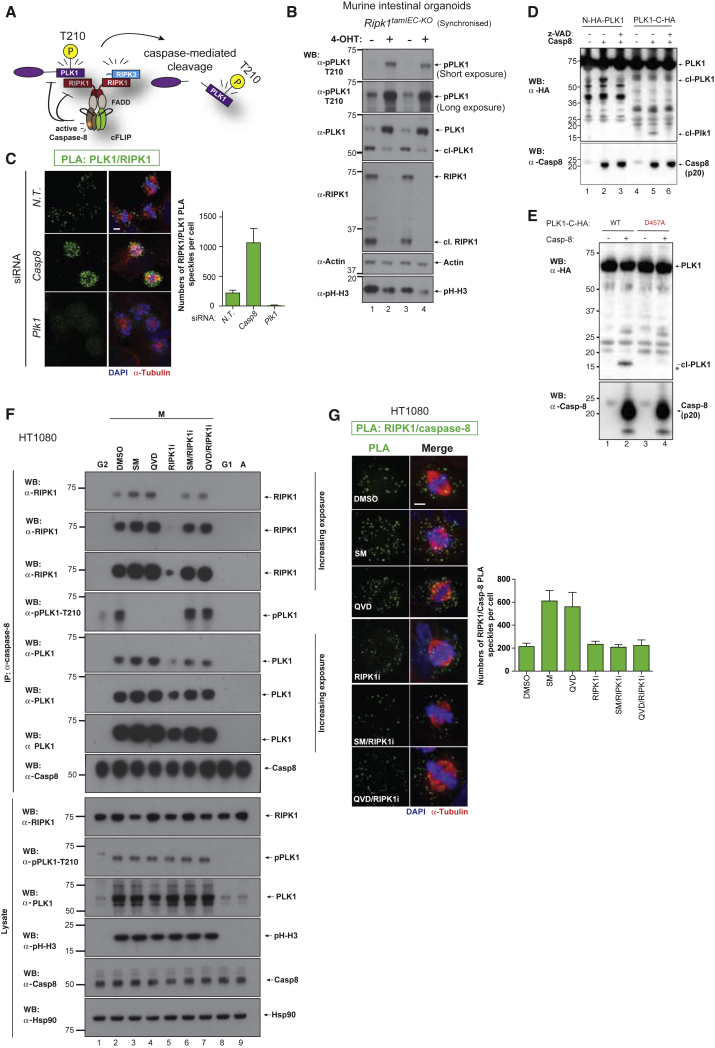
RIPK1 Negatively Regulates PLK1 (A) Schematic representation of RIPK1- and Casp8-mediated regulation of PLK1. (B) Immunoblots of primary intestinal organoids from two *Ripk1*^*fl/fl,IEC-creERTM*^ animals. Organoids were treated with ETOH (vehicle control) or 4-OHT, synchronized with CDK1i, and released into media. Cells were lysed and analyzed by immunoblotting with the indicated antibodies. (C) *In situ* PLA detection of PLK1 and RIPK1 in HT1080 cells, following *Casp8* or *Plk1* siRNA. Green dots indicate PLA signals between PLK1 and RIPK1. Graph shows quantifications of RIPK1/PLK1 PLA speckles (mean ± SD). 15 cells were counted for each condition. Scale bars: 10 μm. (D and E) *In vitro* cleavage assay. Purified HA-tagged PLK1 construct and recombinant Casp8 were incubated for 1 h. Immunoblots analysis using the indicated antibodies is shown. (F) HT1080 cells were synchronized with CDK1i and released in media containing the indicated drugs. Lysates from asynchronous or synchronized HT1080 cells were immunoprecipitated with anti-Casp8 antibody. Immunoblot analysis using the indicated antibodies is shown. (G) *In situ* PLA detection of Casp8/RIPK1 in CDK1i-synchronized and released HT1080 cells treated with the indicated agents. Green dots indicate PLA speckles. Scale bars: 10 μm.

Casp8 can cleave RIPK1, RIPK3, and cFLIP_L_ within ripoptosome complexes (Oberst et al., 2011). Since PLK1 is also recruited to such complexes during mitosis, we tested whether Casp8 cleaves PLK1 in a RIPK1-dependent manner (Figure 3A). Using CDK1i-synchronized and released primary *Ripk1*^*tamIEC-KO*^ intestinal organoids, we noticed that RIPK1 and PLK1 were readily cleaved (Figure 3B). PLK1 cleavage was RIPK1-dependent, as conditional ablation of *Ripk1* caused accumulation of full-length PLK1 while diminishing the cleaved form. *Ripk1* deletion also resulted in enrichment of active PLK1 (PLK1-pT210). Similar results were observed in synchronized Mouse Dermal-Fibroblasts (MDFs) where expression of RIPK1 was ablated by CRISPR/Cas9 (Figure S3A). Depletion of Casp8 resulted in a stronger PLK1/RIPK1 PLA signal (Figure 3C), suggesting that Casp8 negatively regulates PLK1/RIPK1 interactions.

To gain further insights on how Casp8 regulates PLK1, we mapped the Casp8 cleavage site of PLK1. To this end, we purified N- or C-terminally HA-tagged PLK1 from cells (Figure S3B) and offered it to recombinant Casp8 in presence or absence of zVAD-fmk. This revealed the presence of a Casp8 cleavage site (Figures 3D and 3E). Mutagenesis of putative cleavage sites revealed that Casp8 cleaves PLK1 at D457, which is surface exposed and evolutionarily conserved from flies to human (Figures 3D, 3E, and S3B). Together, these data suggest that recruitment of PLK1 to ripoptosome complexes induces Casp8-mediated PLK1 cleavage in a RIPK1-dependent manner and that active PLK1, similarly to RIPK1, is regulated via Casp8-mediated proteolysis.

To further study how the activity and size of mitotic ripoptosome affect PLK1, we decided to utilize pharmacological compounds known to modulate the activity and formation of the ripoptosome (Figure S3C) (Silke and Meier, 2013, Tenev et al., 2011). Pharmacological inhibition of IAPs (via SMAC mimetics, SM), like treatment with the caspase inhibitor QVD, resulted in an increase in ripoptosome formation during mitosis, which was accompanied with enhanced PLK1 recruitment (Figures 3F and 3G). Co-treatment with the RIPK1 inhibitor GSK’963 (Berger et al., 2015) (subsequently referred to as RIPK1i) normalized complex formation (Figures 3F and 3G). While treatment with SM or QVD diminished the recruitment of active PLK1 (pPLK1-T210) into ripoptosome complexes, co-treatment with RIPK1i normalized this interaction (Figures 3F and S3D–S3G). Interestingly, treatment with the RIPK1i alone significantly reduced the association of RIPK1 and PLK1 to the complex, albeit it did not completely block their recruitment to Casp8 (Figure 3F). While the immunoprecipitation in Figure 3F appears to contradict the PLA-based results (Figure 3G), it should be noted that PLA only assesses whether RIPK1 is in proximity to Casp8 but cannot assess how many molecules of RIPK1 are in proximity to Casp8. Given that the same number of PLA-speckles is formed following release into DMSO and RIPK1i, but significantly more RIPK1 co-purifies with caspase-8 under IP/Western conditions, we conclude that the RIPK1 inhibitor suppresses the “chaining” of RIPK1 within the mitotic ripoptosome but does not affect its nucleation. The significant reduction of PLK1 is in line with our observation that recruitment of PLK1 is dependent (and directly proportional) to the recruitment of RIPK1 (Figure 2E). Together, our data are consistent with the notion that enhanced ripoptosome formation negatively impacts PLK1.

### The Ripoptosome Negatively Regulates PLK1-Mediated Phosphorylation of its Downstream Substrates

**Figure 4:**
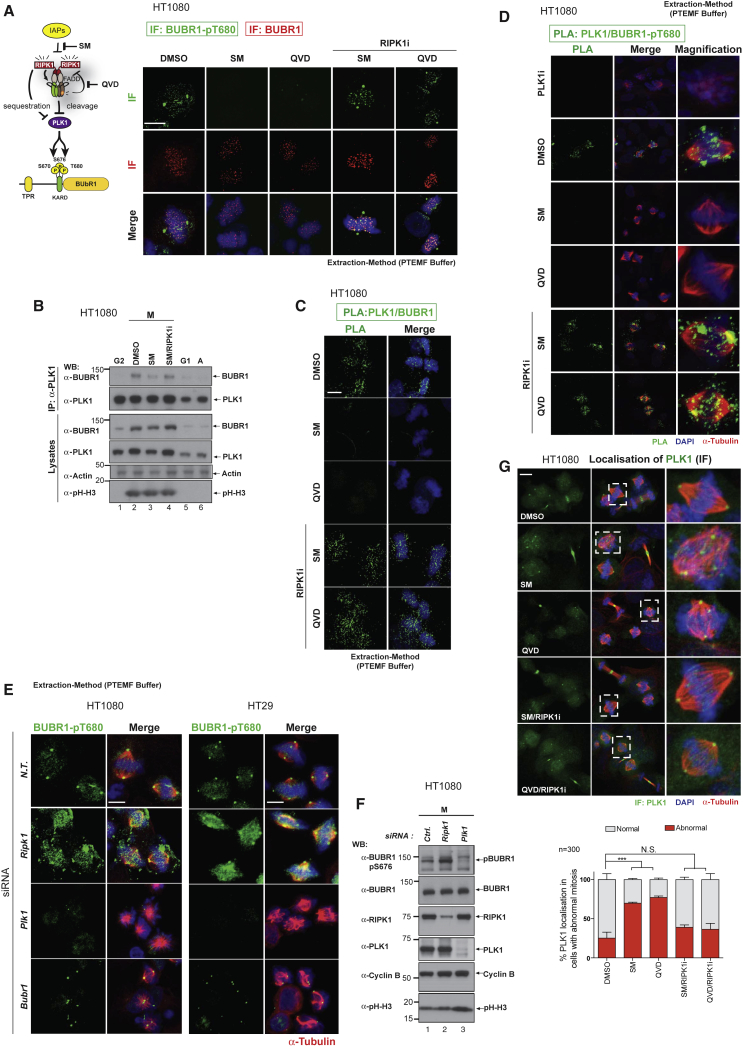
RIPK1 Negatively Regulates PLK1-Mediated Phosphorylation of BUBR1 (A) Scheme illustrating how mitotic ripoptosome interacts and modulates PLK1 and how pharmacological inhibition regulates such interaction and downstream substrate activation. Immunofluorescence analysis using anti-BUBR1 or anti-BUBR1-pT680 antibodies. HT1080 cells were synchronized with CDK1i and released into media containing the indicated agents. Scale bars: 10 μm. (B) HT1080 cells were synchronized with CDK1i and released. Lysates from asynchronous or synchronized HT1080 cells were immunoprecipitated with anti-PLK1 antibody. Immunoblot analysis using the indicated antibodies is shown. (C and D) *In situ* PLA detection of PLK1/BUBR1 (C) or PLK1/BUBR1-pT680 (D) in synchronized HT1080 cells, treated with the indicated agents. Scale bars: 10 μm. (E) Immunofluorescence analysis using anti-BUBR1-pT680 antibodies (under extraction conditions) in CDK1i-synchronized HT1080 (left) and HT29 (right) cells under the indicated RNAi conditions. Cells were released for 30 min, after which MG132 was added for 90 min to arrest cells in metaphase. N.T. indicates non-targeting RNAi Control oligos. Scale bars: 10 μm. (F) Western blot analysis of phosphorylated BUBR1 following knockdown of Control (Ctrl), *Ripk1*, or *Plk1* in CDK1i-synchronized and released HT1080 cells. (G) CDK1i-synchronized HT1080 cells were released into media containing the indicated agents. Cells were released for 30 min, after which MG132 was added for 90 min to arrest cells in metaphase. Only cells presenting mitotic abnormalities were scored for PLK1 localization. Images show representative examples of PLK1 mis-localization. Scale bars: 10 μm.

To test whether the ripoptosome negatively regulates PLK1-mediated phosphorylation of its downstream substrates, we monitored the phosphorylation status of BUBR1 (Elowe et al., 2010). PLK1-mediated phosphorylation of BUBR1 is required for chromosome congression and the bipolar spindle attachment that forms the metaphase plate (Suijkerbuijk et al., 2012). We noticed that treatment with SM and QVD caused a dramatic loss of BUBR1 phosphorylation at T680 (Figures 4A, S4A, and S4B). SM and QVD reduced phosphorylation at T680 of BUBR1 to a similar extent as treatment with PLK1i, or following *Plk1* siRNA (Figures S4A and S4B). Importantly, co-treatment with RIPK1i normalized BUBR1 phosphorylation at T680 (Figures 4A and S4B). Consistent with our observed reduction in BUBR1 phosphorylation, we found that treatment with SM caused reduced binding of PLK1 to BUBR1 (Figure 4B). While treatment with SM reduced PLK1-BUBR1 interactions, co-treatment with RIPK1i restored the binding of PLK1 to BUBR1 to control levels. Similar results were obtained by PLA analysis (Figures 4C, 4D, S4C, and S4D). Since the levels of BUBR1 were unchanged following treatment with the respective inhibitors (or inhibitor combinations) (Figures 4A and S4C), our data suggest that deregulation of the ripoptosome interferes with PLK1-mediated interaction and phosphorylation of BUBR1. Consistently, we found that the level of BUBR1 phosphorylation was dramatically increased upon RNAi-mediated depletion of RIPK1 (Figures 4E and 4F). Although ripoptosome modulation affects PLK1-mediated phosphorylation of BUBR1, BUBR1 is unlikely to be the only PLK1 substrate that is affected. This is because treatment with SM and QVD caused mis-localization of PLK1 and abnormal mitosis (Figure 4G). Together, our data demonstrate that enhanced ripoptosome formation negatively impacts PLK1’s ability to phosphorylate downstream substrates such as BUBR1.

### Acute Pharmacological Modulation of RIPK1 or Casp8 Activity, Specifically during Mitosis, Results in Chromosome Alignment Defects

**Figure 5:**
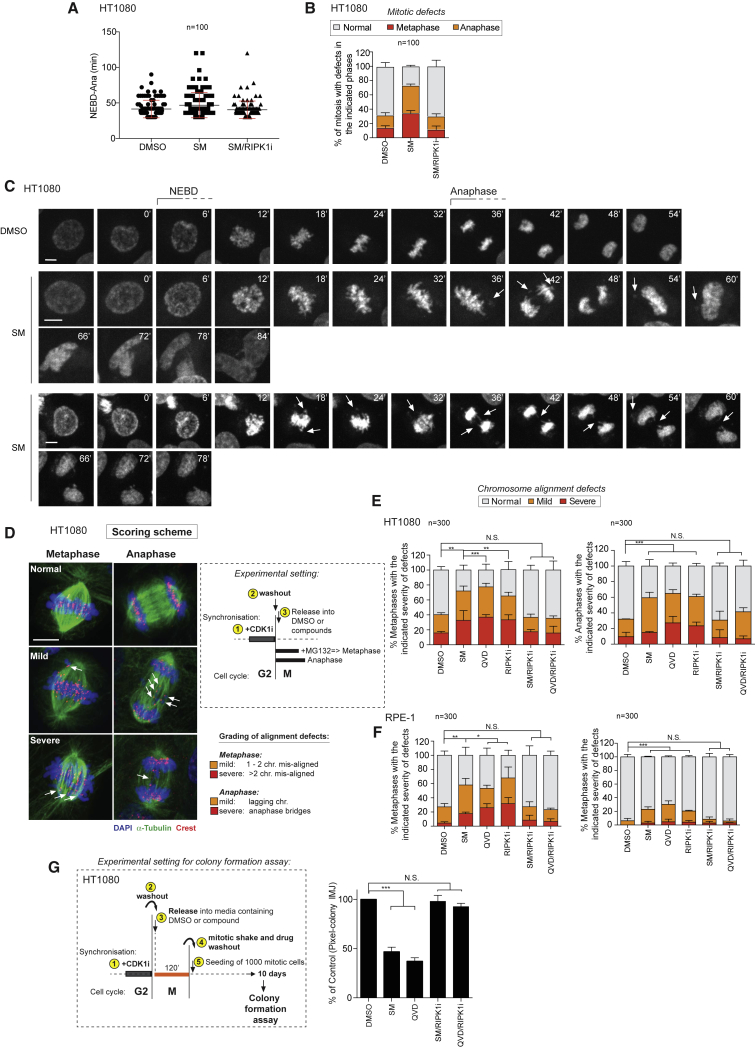
Hyper-Activation of RIPK1 Induces Chromosome Mis-Alignment (A) Asynchronized HT1080 cells were pre-incubated for 2 hr with 10 nM SIR-DNA and then treated with the indicated compounds. Mitotic duration of HT1080 assessed by quantifying the time elapsed between nuclear envelope breakdown (NEBD) and anaphase onset following treatment with the indicated agents. (B) Mitotic abnormalities detected in cells imaged by advanced spinning disc confocal microscopy time lapse to determine mitotic timing. Graphs show the percentage of mitotic abnormalities recorded in 100 mitosis per condition. (C) Example of mitotic cells visualized during advance spinning confocal time lapse. Frames were acquired every 6 min. (D) Scheme depicting experimental procedure for synchronization and release of cells during mitosis in indicated drugs. Examples of chromosome alignment defects to illustrate the scoring system. Scale bars: 10 μm. (E and F) CDK1i-synchronized HT1080 (E) and RPE-1 (F) cells were released into media containing the indicated agents. For the analysis in metaphases, cells were released for 30 min, after which MG132 was added for 90 min to arrest cells in metaphase. Anaphases were scored after 2 hr release. Graphs indicate the number (n) of mitosis scored from 3 independent experiments. Statistical analysis was performed via the two-way ANOVA multiple comparison analysis with ^∗^p < 0.05, ^∗∗^p < 0.01, ^∗∗∗^p < 0.001. Total number of abnormalities was considered in determining statistical significance. Scale bars: 10 μm. (G) Long-term colony formation assay of CDK1i-synchronized HT1080 cells that were released into media containing the indicated drugs for 2 hr. Mitotic cells were collected by shake off, washed, and 1,000 cells were re-plated for clonogenic assay in the absence of drug. Graphs show the mean ± SE of three independent experiments, normalized to control. Two-way ANOVA multiple comparison analysis with ^∗^p < 0.05, ^∗∗^p < 0.01, ^∗∗∗^p < 0.001.

We next tested whether RIPK1-mediated deregulation of PLK1 results in mitosis defects. First, we evaluated mitotic timing via live-cell imaging. While there was no significant difference in the length of mitosis of cells treated with DMSO, SM, or SM/RIPK1i (Figure 5A; Videos S1, S2, and S3), careful analysis of individual frames revealed that SM-treated cells presented accumulation of chromosomal defects during both metaphase and/or anaphase (Figures 5B, 5C, and S5A; Videos S1, S2, and S3). Interestingly, these defects were completely rescued by co-treatment with RIPK1i, which is in agreement with our observation that RIPK1i restores defects in BUBR1-pT680 phosphorylation upon SM treatment (Figures 4A, 4B, and 4D–4G).

Video S1. Live Cell Imaging of HT1080 Cells Treated with DMSO, Related to Figure 5 Asynchronised HT1080 cells were pre-incubated for two hr with 10 nM SIR-DNA and then treated with DMSO. Live cell imaging was recorded by advance spinning confocal time lapse filming. Frames were acquired every 6 min for 10 hr. Only the first 5 hr (90 frames) were taken into consideration. Movies should be opened via ImageJ and color balance should be adjusted according to the user preferences.

Video S2. Live Cell Imaging of HT1080 Cells Treated with SM, Related to Figure 5 Asynchronised HT1080 cells were pre-incubated for two hr with 10 nM SIR-DNA and then treated with SM. Live cell imaging was recorded by advance spinning confocal time lapse filming. Frames were acquired every 6 min for 10 hr. Only the first 5 hr (90 frames) were taken in consideration. Movies should be opened via ImageJ and color balance should be adjusted according to the user preferences.

Video S3. Live Cell Imaging of HT1080 Cells Treated with SM and RIPK1i, Related to Figure 5 Asynchronised HT1080 cells were pre-incubated for two hr with 10 nM SIR-DNA and then treated with SM/RIPK1i. Live cell imaging was recorded by advance spinning confocal time lapse filming. Frames were acquired every 6 min for 10 hr. Only the first 5 hr (90 frames) were taken in consideration. Movies should be opened via ImageJ and color balance should be adjusted according to the user preferences.

To gain further insights on the type of chromosomal abnormalities, we evaluated fixed mitotic cells released into media containing MG132, which allows accurate evaluation of chromosome segregation defects (Janssen et al., 2009) (Figure 5D). Microscopic images of mitotic HT1080 and RPE-1 cells, treated with SM, QVD, zVAD, or RIPK1i, revealed a significantly higher number of chromosome alignment defects at the metaphase plate and abnormalities at anaphase (Figures 5E, 5F, and S5B–S5F). Importantly, the SM- and QVD/zVAD-mediated chromosomal abnormalities were fully rescued upon co-treatment with two different RIPK1 kinase inhibitors, RIPK1i and Nec-1s (Figures 5E, 5F, and S5C–S5F). In agreement with our data from HT1080 cells (Figure S5G), analysis of synchronized RPE-1 cells also showed that ripoptosome modulation caused the appearance of multipolar spindles (Figure S5H). This is consistent with a role of PLK1 in regulating Kizuna, TPX2, chTOG, TACC3, and clathrin (Maiato and Logarinho, 2014). Despite the abundance of such abnormalities, cells seemed to be able to complete mitosis (on average at the same time), though in some cases without achieving daughter cell separation. Nevertheless, this had significant consequences as treatment with SM, QVD, or RIPK1i led to a significant drop in clonogenic potential, which was restored upon co-treatment with the RIPK1 inhibitors RIPK1i and Nec-1s (Figures 5G and S5I). No such effect was observed when cells were acutely (2 hr) treated with these compounds outside of M-phase (Figure S5J). Together, these data strongly suggest that RIPK1 and Casp8 contribute to proper chromosome dynamics during mitosis and that their deregulation can result in chromosome alignment defects.

### Cells from *Ripk1* and *Casp8* Knockout Animals Exhibit Chromosome Alignment Defects

**Figure 6:**
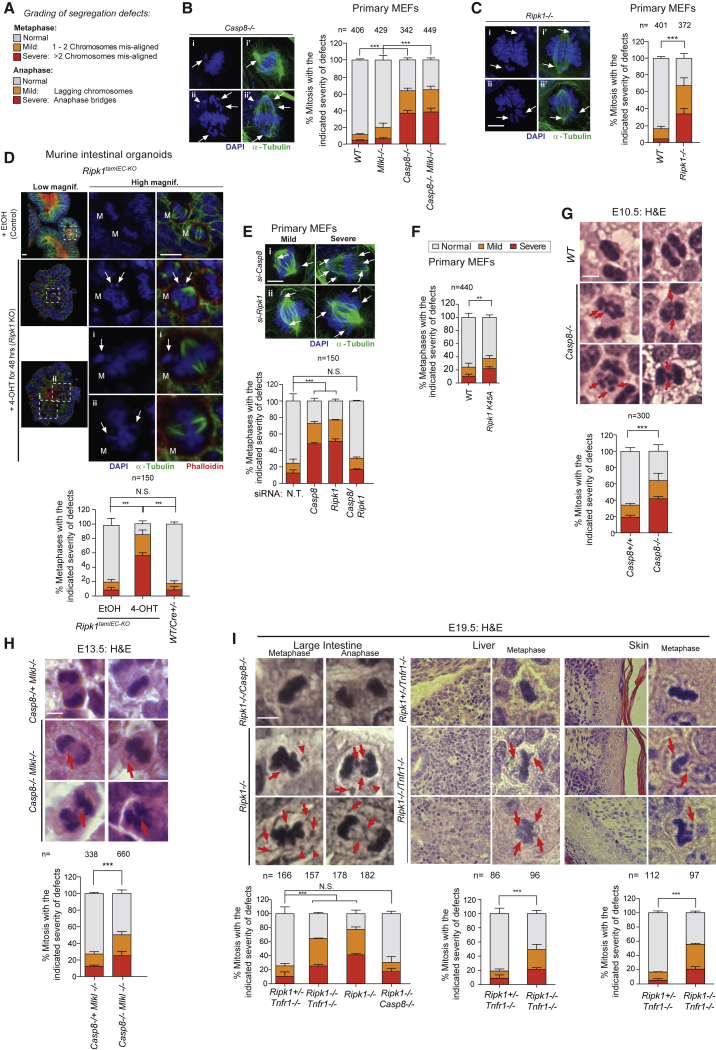
Cells from *Ripk1* and *Casp8* Knockout Animals Harbor Defects in Chromosome Alignment (A) Grading of segregation defects. (B and C) Chromosome alignment defects of the indicated primary MEFs. Images show representative phenotypes. Cells were released for 30 min after which 10 μM MG132 was added for 90 min to arrest cells in metaphase. Graphs indicate the number (n) of mitosis scored from 3 independent experiments. Statistical analysis was performed via the two-way ANOVA multiple comparison analysis with ^∗^p < 0.05, ^∗∗^p < 0.01, ^∗∗∗^p < 0.001. Total amount of abnormalities was considered in determining statistical significance Scale bars: 10 μm. (D) Primary intestinal organoids from *Ripk1*^*fl/fl,IEC-creERTM*^ animals. Organoids were treated with ETOH (vehicle control) or 4-OHT, synchronized with CDK1i, released into media, and scored for alignment defects. Cells were released for 30 min, after which 10 μM MG132 was added for 90 min to arrest cells in metaphase. Graphs indicate the number (n) of mitosis scored from 3 independent experiments. Statistical analysis was performed via the two-way ANOVA multiple comparison analysis with ^∗^p < 0.05, ^∗∗^p < 0.01, ^∗∗∗^p < 0.001. Arrows indicate misaligned chromosomes. Total amount of abnormalities was considered in determining statistical significance. Scale bars: 10 μm. (E) Chromosome alignment defects of the indicated primary MEFs following knockdown of indicated genes. Images show representative phenotypes. Cells were released for 30 min after which 10 μM MG132 was added for 90 min to arrest cells in metaphase. Graphs indicate the number (n) of mitosis scored from 3 independent experiments. Statistical analysis was performed via the two-way ANOVA multiple comparison analysis with ^∗^p < 0.05, ^∗∗^p < 0.01, ^∗∗∗^p < 0.001. Total amount of abnormalities was considered in determining statistical significance Scale bars: 10 μm. (F) Chromosome alignment defects of the indicated primary MEFs following knockdown of indicated genes. Images show representative phenotypes. Cells were released for 30 min after which 10 μM MG132 was added for 90 min to arrest cells in metaphase. Graphs indicate the number (n) of mitosis scored from 3 independent experiments. Statistical analysis was performed via the two-way ANOVA multiple comparison analysis with ^∗^p < 0.05, ^∗∗^p < 0.01, ^∗∗∗^p < 0.001. Total amount of abnormalities was considered in determining statistical significance Scale bars: 10 μm. (G–I) Hemotoxylin- and eosin-stained sections of embryos of the indicated age and genotypes. Mitotic abnormalities were scored throughout the entire embryo (G and H) and the large intestine, liver and skin (I). The graph indicates the SE of mitotic abnormalities scored from three (G and H) and two embryos (I). Total amount of abnormalities was considered in determining statistical significance.

Next, we wished to characterize cells from available knockout animals to determine whether genetic deletion of ripoptosome components results in chromosome alignment defects. To this end, we assessed chromosomal segregation defects in synchronized primary MEFs derived from *WT*, *Casp8*^*fl/fl*^ *RosaCreER (Casp8*^*−/−*^ MEFs were obtained following treatment with Tamoxifen), *Ripk1*^*−/−*^, *Mlkl*^*−/−*^, and *Casp8/Mlkl double-knockout (DKO)* mice using a blinded-experimental design (Figures 6A–6C). Knockout cell lines were validated functionally and via western blot (Figures S6A–S6E). Our data showed that 63% of *Casp8*^*−/−*^ mitotic cells had chromosome segregation defects (Figure 6B). Importantly, co-deletion of *Mlkl* did not rescue these defects, as 64% of *Casp8*/*Mlkl* DKO MEFs exhibited chromosome alignment defects (Figure 6B). This demonstrates that the alignment defects of primary *Casp8*^*−/−*^ MEFs occur independently of the necroptotic program. Intriguingly, similar to *Casp8*^*−/−*^ MEFs, 66% of primary *Ripk1*^*−/−*^ MEFs displayed chromosome alignment defects (Figure 6C). Since *Ripk1*^*−/−*^ *and Casp8*^*−/−*^ *cells* exhibited comparable levels of chromosome alignment defects, these data suggest that RIPK1 and Casp8 together contribute to proper chromosome alignment. The observed chromosome alignment defects were also evident in conditional *Ripk1*^*tamIEC-KO*^ intestinal organoids (Figures 6D and S6F–S6J) but were absent in EtOH-treated *WT* organoids. Importantly, 4-OHT-treated *Cre* control organoids also did not present any abnormalities, indicating that the observed chromosomal defects in *Ripk1*^*tamIEC-KO*^ were due to the conditional deletion of RIPK1 (Figures 6D and S6G–S6J). Like genetic deletion, RNAi-mediated knockdown of *Ripk1* or *Casp8* also caused chromosome misalignment in primary MEFs and human HT1080 cells (Figures 6E, S6K, S6L, and data not shown). Knockdown efficiency was determined functionally (Figure S6K) and biochemically (Figure S6L). Interestingly, co-knockdown of *Casp8* and *Ripk1* rescued chromosome alignment defects in primary MEFs (Figure 6E), which is in agreement with our pharmacological study using QVD and RIPK1i. Primary MEFs derived from *Ripk1*^*K45A*^ knockin mice showed modest chromosome alignment defects compared to control. However, these were numerically lower than those observed in *Ripk1*^*−/−*^ (Figure 6F).

Chromosome alignment defects were also apparent in tissue sections derived from *Casp8*^*−/−*^ (64%) (E10.5), *Casp8/Mlkl DKO* (50%) (E13.5), *Ripk1*^*−/−*^ (large intestine 78%, liver 49%, and skin 55%) (E19.5), and *Ripk1/Tnfr1 DKO* (large intestine 64%, liver 49%, and skin 55%) (E19.5) but were absent in tissue sections from *Ripk1/Casp8* DKO (E19.5) embryos or *Mlkl*^*−/−*^ (E13.5) embryos (Figures 6G, 6H, and 6I). Together, this demonstrates that RIPK1 and Casp8 are involved in regulating chromosome segregation, independently of their role in inhibiting necroptosis or inflammation. While 78% of mitotic cells in the large intestine of *Ripk1*^*−/−*^ embryos had chromosome segregation defects, co-deletion of *Casp8* completely rescued the *Ripk1*^*−/−*^ segregation defects at E19.5 (Figure 6I). In contrast, co-deletion of *Tnfr1* did not rescue the alignment defects of *Ripk1*^*−/−*^ animals, even though it did rescue caspase-3 activation and apoptosis in the large intestine and liver (Figure S6M; Rickard et al., 2014). This demonstrates that cell death and chromosome alignment defects can be uncoupled genetically and provides further evidence for a non-cell death function of RIPK1 and the ripoptosome in mitosis.

### *Ripk1* mRNA Levels Correlate with Aneuploidy in Human Cancers

**Figure 7:**
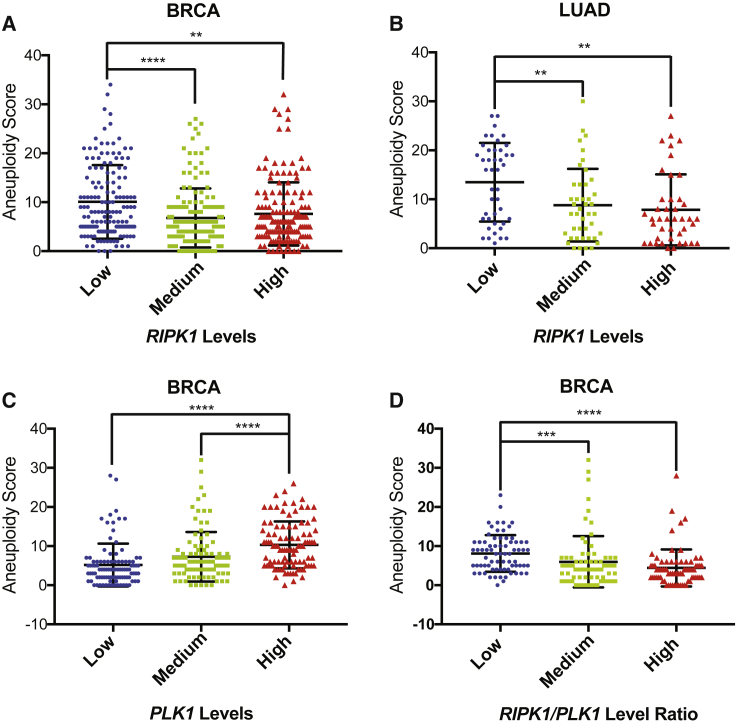
*Ripk1* mRNA Levels Correlate with Aneuploidy in Human Cancers (A–D) Bioinformatics analyses of aneuploidy scores in association with *RIPK1* mRNA expression (A–B), *PLK1* mRNA expression (C), or the normalized ratio of the two (D) in breast (A, C, D) and lung (B) cancer patients. BRCA: breast cancer; LUAD: lung adenocarcinoma. ^∗∗^p < 0.01; ^∗∗∗^p < 0.001; ^∗∗∗∗^p < 0.0001. (A) *n*: 461; (B) *n*: 128; (C) *n*: 293; (D) *n*: 218.

Chromosomal mis-segregation defects are known to induce aneuploidy, which in turn has been shown to correlate with tumor onset and progression, but also therapeutic treatment (Bakhoum and Swanton, 2014). Recently, a score of aneuploidy was calculated for most tumor samples, accessible by The Cancer Genome Atlas (TCGA) (Taylor et al., 2018), allowing us to correlate gene expression to aneuploidy. Our results clearly indicate that low expression of *RIPK1* mRNA is significantly associated with higher aneuploidy in breast, lung, and colorectal carcinomas (Figures 7A, 7B, and S7A). Our analysis excluded samples bearing *TP53* mutations (Taylor et al., 2018) and copy number variations of the *RIPK1* gene. Also, we utilized *CENP-A* (centromeric histone) and *PLK1* as positive controls (Taylor et al., 2018). As expected, tumors with high levels of CENP-A or *PLK1* displayed higher aneuploidy (Figures 7C, S7B, and S7C). Interestingly, a low ratio between normalized expression of *RIPK1* and *PLK1* mRNA levels showed an association with higher aneuploidy (Figure 7D). Together, these data suggest that a combination of low RIPK1 and high PLK1 might be associated with chromosomal mis-segregation and long-term aneuploidy in human cancers.

## Discussion

RIPK1 and Caspase-8 are key players in inflammation, cell death, and the regulation of tissue homeostasis, positively and negatively regulating these processes depending on the cellular context (Pasparakis and Vandenabeele, 2015). Beyond their role in inflammation and cell death, we find that RIPK1 and Casp8 also play critical roles in mitosis.

Several lines of evidence demonstrate that RIPK1 and Casp8 contribute to normal mitosis. First, in mouse and human, the ripoptosome naturally forms during mitosis, peaking at metaphases and disassembling as cells exit M-phase. Consistently, mitotic cells harbor sub-lethal levels of caspase activity that is dependent on RIPK1, Casp8, and cFLIP. Second, PLK1 is incorporated into the ripoptosome specifically in mitosis, an observation that is consistent with previous reports demonstrating an interaction between PLK1 and Casp8 (Matthess et al., 2014) and FADD (Jang et al., 2011). Like for RIPK1 and RIPK3, recruitment of PLK1 to the ripoptosome results in Casp8-mediated cleavage. Third, ripoptosome-mediated regulation of PLK1 fine-tunes the localization and activity of PLK1. Downregulation and hyper-activation of ripoptosomes cause defects in PLK1 localization, PLK1-BUBR1 interaction, and loss of PLK1-mediated phosphorylation of BUBR1 (and most likely other PLK1 substrates), resulting in mitotic defects. Fourth, genetic deficiency or mitosis-specific pharmacological inhibition of ripoptosome components causes severe chromosomal alignment defects. These defects occur independently of cell death or inflammation. This is evident as *Casp8/Mlkl DKO* mice still present chromosomal instability, despite rescuing necroptotic cell death. Likewise, *Ripk1/Tnfr1 DKO* animals, which are fully viable at E19.5, harbor significant levels of chromosome alignment defects in various tissues in the absence of caspase-3 activation. Hyper-formation of mitotic ripoptosomes, following SM treatment, may possibly inhibit PLK1 function by a two-pronged mechanism: (1) via proteolytic cleavage of PLK1 within ripoptosome complexes and (2) by recruitment of PLK1 into ripoptosome complexes and sequestration away from its substrates. Together, these reduce the availability of active PLK1 to phosphorylate important downstream substrates required for chromosome alignment and congression. Consistent with a role of sequestration, *Casp8* knockdown enhances the recruitment of PLK1 to RIPK1. Conversely, loss of RIPK1 de-represses PLK1, leading to substrate hyper-phosphorylation. Our current model proposes that PLK1 is recruited into the ripoptosome complexes via RIPK1 and that RIPK1 negatively regulates PLK1. This interaction is further regulated by Casp8, which cleaves not only RIPK1 but also PLK1 at D457. The cleavage of RIPK1 is required to regulate the recruitment of PLK1 and keep its activity under tight regulation. Upon loss of Casp8 or IAP depletion, the RIPK1-PLK1 association is enhanced leading to sequestration and inactivation of PLK1, ultimately interfering with PLK1-mediated phosphorylation of BUBR1. Under this condition, deregulated RIPK1 might bind to PLK1 and sequester it away from the spindle or chromosomes. On the other hand, when RIPK1 is missing, Casp8-mediated cleavage of PLK1 is lost (Figures 3B and S3A), leading to accumulation of active PLK1. However, this alone would not explain why co-deletion of *Ripk1/Casp8* normalizes the chromosomal instability observed in absence of either Casp8 or RIPK1. It is possible that, in the absence of *Ripk1*, Casp8 might (indirectly) lead to PLK1 over-activation, potentially by cleaving and inactivating a protein involved in PLK1 regulation at the spindle. Hence, concomitant loss of both RIPK1 and Casp8 would not only prevent RIPK1-induced inhibition of PLK1 but also Casp8-mediated over-activation of PLK1, leaving levels of PLK1 intact.

Mice with a *Ripk1 kinase-dead* knock-in-allele (*Ripk1*^*K45A*^) develop normally (Berger et al., 2014), even though primary MEFs of such animals do have some chromosome alignment defects, although such defects are very modest when compared to *Ripk1*^−/−^. Similar chromosome alignment defects, as well as aneuploidy, are also seen in *Bubr1*^*−/+*^ mice, yet such mice are fertile and die of old age and present only splenomegaly and abnormal megakaryopoiesis (Baker et al., 2004, Kapanidou et al., 2015). The tumorigenic phenotype due to this lingering chromosomal instability is manifested only upon challenge with the drug azoxymethane (Dai et al., 2004). It is, therefore, possible that the moderate number of chromosomal defects observed in the *Ripk1*^*kd*^ is tolerated by the animals without affecting survival or fertility.

Intriguingly, the mitotic ripoptosome seems to differ from other situations where ripoptosome/complex-II/necrosome complexes are formed. While depletion of cIAPs, activation of cytokine, or pattern recognition receptors results in RIPK1-driven formation of RIPK1/FADD/Casp8/cFLIP complexes, during mitosis the formation of this complex seems to be driven by either FADD or Casp8. This is evident as FADD/Casp8 complexes form independently of RIPK1. Further, while RIPK1 kinase inhibition blocks “canonical” ripoptosome formation, treatment with RIPK1i only prevents chaining of RIPK1 onto the mitotic ripoptosome without affecting the nucleation of the complex. This suggests an important difference between kinase activity of RIPK1 and scaffolding function of RIPK1, both equally important for the regulation of PLK1. This is especially highlighted by the fact that loss of RIPK1 kinase activity in animals only induces low levels of chromosomal instability, a phenotype somewhat intermediate to the loss of RIPK1.

Our findings that the ripoptosome contributes to mitosis are consistent with earlier reports demonstrating that Casp8 is essential for maintaining chromosome stability, suppressing B cell lymphomagenesis (Hakem et al., 2012), restraining oncogene-induced transformation (Krelin et al., 2008), and acting as driver mutation in breast cancer (Stephens et al., 2012); also, it is frequently found to be mutated or lost in different cancer types (Hopkins-Donaldson et al., 2003, Martinez et al., 2007, Soung et al., 2005b). This, together with our analysis that mRNA levels of *RIPK1* are associated with aneuploidy in breast, lung, and colon cancer, strongly suggests that Casp8- and RIPK1-mediated regulation of PLK1 is required for the maintenance of chromosome stability, consequent aneuploidy, and suppression of cancer (at least in the B cell lineage for Casp8).

Our observations have important ramifications, as they may help to explain why *Casp8* is frequently lost in several kinds of human tumors, including small-cell lung carcinoma, neuroblastoma, hepatocellular carcinoma, and others. Interestingly, *Casp8* deficiency occurs by multiple mechanisms, including hyper-methylation of regulatory regions in the *Casp8* gene or (less frequently) gene mutation, suggesting that it is a causal factor in the oncogenic transformation rather than a consequence of it. Consistently, our data suggest that loss of Casp8 and subsequent deregulation of RIPK1 would provide cells with evolvability (chromosomal instability), leading to establishment of cellular transformation and tumorigenesis.

## STAR★Methods

### Key Resources Table

**Table:** 

REAGENT or RESOURCE	SOURCE	IDENTIFIER
**Antibodiess**		

Anti-RIPK1 (C-terminal) – for IP western and PLA	BD Bioscience	610459
Anti-PLK1 for Human WB, PLA, IF	Bethyl Laboratories	A300-251A
Anti-PLK1 for Mouse WB	Abcam	Ab178666
Anti-pPLK1-T210 for WB, PLA, IF	Abcam	AB155095
Anti-Actin for WB	Santa Cruz Biotechnology	sc-1615
Anti-BUBR1 for WB, IF, PLA	BD bioscience	8242
Anti-BUBR1-pT680 for IF	Gert Kops	N/A
Anti-BUBR1-pS676 WB	Erich A. Nigg	sc-371
Anti-p-H-H3 for WB	Millipore	06-570
Anti-cFLIP WB PLA	Adipogene	AG-20B-0056
Anti-MYC for IP and WB	SIGMA	M5546
Anti-RIPK3 – Human WB PLA	Novus Biological	NBP2-24588
Anti-RIPK3 – Mouse WB PLA	ProSci Inc	2283
Anti-FADD – for IP and PLA	Santa Cruz Biotechnology	sc-6036
Anti-FADD – for WB	BD	610400
Anti-RIPK1 (N-terminal) WB PLA	Cell Signaling	3493
Anti- phalloidin633 IF	Invitrogen	A22284
Anti-alpha-Tubulin IF	Serotec	mca78g
Anti-CASPASE-8 - for WB - post IP Human	MBL	M032-3
Anti-CASPASE-8 - for IP [C-20] Human	Santa Cruz Biotechnology	sc-6136
Anti-HA For IP	Roche	11867423001
Anti-HSP90 WB	Santa Cruz Biotechnology	sc-7947
Anti-PLK1 for Mouse WB and PLA		AB178666
Anti- Casp8 for Mouse PLA	Santa Cruz Biotechnology	SC7890
Anti Crest for IF	Kind Gift of Stephan Geley	N/A
Donkey anti- rat 488	Invitrogen	A21208
Donkey anti-rat 633	Biotium	20137
Goat anti- human 555	Invitrogen	A21433
Goat anti- rabbit 488	Invitrogen	A11034
Donkey anti-mouse 488	Invitrogen	A21202
Goat anti- rabbit 633	Invitrogen	A21071
Anti- alpha – tubulin 488	Invitrogen	322588
Anti-goat plus	SIGMA	DUO 82003
Anti-mouse minus	SIGMA	DUO 82004
Anti-mouse plus	SIGMA	DUO 92004
Anti-rabbit plus	SIGMA	DUO 82002
Anti-Rabbit Minus	SIGMA	DUO 82005

**Chemicals, Peptides, and Recombinant Proteins**		

Mouse recombinant TNF	Enzo Life Sciences	ALX-522-009-C050
Smac Mimetic (164)	Gift from X. Wang	N/A
GSK’963 (RIPK1 inhibitor)	Gift from GSK	N/A
cOmplete EDTA-free protease inhibitor tablets	Roche	11873580001
Halt Protease and phosphatase inhibitor	Thermo Scientific	78443
QVD	Apex Bio	A1901
zVAD-FMK	Apex Bio	A1902
Protein G Sepharose	Sigma	P3296
Dapi	Invitrogen	DB571
Propidium iodide solution	Sigma	P4864
Ac-DEVD-AMC	Sigma	A1086
MG132	Sigma	C2211
CDK1i (RO3306)	Tocris	4181
Bim Peptide	Kind Gift of Tony Letai	N/A
Nec1	Merck	CAS-4311-880
Nec-1s	Biovision	2263
Recombinant Casp-8	Enzo	Axl-201-062-0100
Prolong Gold	Invitrogen	P36934
SirDNA	Spirochrome	SC007
h-TNF	Enzo Life Sciences	ALX-804-034-C050

**Critical Commercial Assays**		

Duolink In Situ Detection Reagents Green	Sigma	DUO92014

**Deposited Data**		

Raw data	http://doi:10.17632/x8tvmp6rfb.1	N/A

**Experimental Models: Cell lines**		

Kym1	Gift from John Silke (Melbourne, Australia)	N/A
HT1080	ATCC	CCL-121
MDA-MB-231	In house	N/A
HEK293T	In house	N/A
Flp-In™T-REx™-HEK293	Termo Scientific	R78007
HT29	In house	N/A
RPE-1	In house	N/A
U2OS	In house	N/A
Primary MEFs	In house	N/A
T47D	In house	N/A
Primary MEFs *Ripk1*^*-/-*^	Gift from Manolis Pasparakis	N/A
Primary MEFs *Ripk1*^*+/+*^	Gift from Manolis Pasparakis	N/A
PrimaryMEFs *Casp8*^*fl/fl*^	Gift From Andrew Oberst	N/A
Primary MEFs *Casp8*^*-/-*^*Mlkl*^*-/-*^	Gift From Andrew Oberst	N/A
MDFs	In house	N/A
MDFs *Ripk3*^*-/-*^	In house	N/A
Intestinal organoids *Ripk1*^*fl/fl,IEC-creERTM*^	Gift from Manolis Pasparakis	N/A
MEFs *Ripk1*^*k45A*^	In house	N/A

**Experimental Models: Organisms/Strains**		

Mouse: C57BL/6 *Ripk1*^*-/-*^	Gift from John Silke	N/A
Mouse: C57BL/6 *Ripk1*^*-/-*^*Tnfr1*^*-/-*^	Gift from John Silke	N/A
Mouse: C57BL/6 *Mlkl*^*-/-*^	Gift from Henning Walczak	N/A
Mouse: C57BL/6 *Ripk1*^*-/-*^*Casp8*^*-/-*^	Gift from Henning Walczak	N/A
Mouse: C57BL/6 *Ripk1*^*/+-*^*Tnfr1*^*-/-*^	Gift from John Silke	N/A

**Recombinant DNA**		

pcDNA3	Thermo Scientific	V79020

**Software and Algorithms**		

GraphPad Prism v6.0	http://www.graphpad.com/	N/A
Imaris 8.0	Imaris Ltd	N/A
Image J	https://imagej.nih.gov/ij/	N/A
FACs DIVA		N/A

### Contact for Reagent and Resource Sharing

Further information and requests for resources and reagents should be directed to and will be fulfilled by the Lead Contact, Pascal Meier (p.meier@icr.ac.uk).

### Experimental Model and Subject Details

#### Experimental Model

All animal procedures were conducted in accordance with the guidelines of The Walter and Eliza Hall Institute Animal Ethics Committee, UK home office in accordance with the revised (2013) Animals (Scientific Procedures) Act (ASPA) and the institutional guidelines of the UCL Cancer Institute, or European, national and institutional guidelines approved by the local government authorities of the Landesamt fuer Natur, Umwelt und Verbraucherschutz Nordrhein-Westfalen, Germany.

#### Cell lines

Cell lines were obtained from ATCC (American Type Culture Collection). HT1080, RPE-1, KYM1, MDA-MB-231, T47D, ZR75, primary MEFs (passage number 2), HEK293T, U20S were cultured in DMEM. Culture media were supplemented with 10% fetal bovine serum (GIBCO), penicillin, and streptomycin. Cells were cultured under 10% CO_2_.

### Method Details

#### Isolation of Primary cells

Primary Mouse Embryonic Fibroblasts (MEFs) were generated from E13.5 embryos. After removing the placenta, yolk sac, head and the dark red organs, embryos were finely minced and digested for 20 min in 0.25% trypsin. Single cell suspension was then obtained by pipetting up and down the digested embryos. Mouse Dermal Fibroblasts (MDFs) were isolated as described in (Etemadi et al., 2015). To generate Bone Marrow Derived Macrophages (BMDMs), bone marrow cells from tibia and femur of 2 month old mice were seeded in non-coated Petri dishes and cultured for 6 days in Dulbecco’s modified Eagle medium + 10% fetal bovine serum + 20% (v/v) L929 mouse fibroblast conditioned medium. Keratinocytes were isolated as described in (Lichti et al., 2008). Splenocytes were isolated from 2 month old mice. Mouse spleens were mashed through a cell strainer into the Petri dish using the plunger end of a syringe. Cells were then washed once in cold PBS and treated with 1X Red Blood Cell Lysis Buffer (BioLegend, Cat N 420301) for 5 min on ice. Cells were then washed again in PBS and counted.

#### Isolation of primary intestinal organoids

Small intestine crypts were isolated and grown as described previously (Dannappel et al., 2014). Deletion of *Ripk1* in organoid cultures from *Ripk1*^*fl/fl,IEC-creERTM*^ (RIPK1^tamIEC-KO^) was induced with 100 nM 4-OHT for 24 hr. *Ripk1*^*fl/fl,IEC-creERTM*^ treated with ETOH for the duration of 24 hr were used as controls. To synchronize organoid cultures, cells were treated with 9 μM CDK1i for 20 hr, after which cells were released using 3x washes with media. 10 min after release, organoid cultures were supplemented with 20 μM MG132 for 90 min. Chromosome misalignment was assessed 48 hr and 72 hr after 4-OHT treatment. For western blot analysis: intestinal organoids were harvested, and passed through a 23 G syringe. Cells were centrifuged at 10000 rpm for 5 min. Pelleted organoids were resuspended in 80 μl RIPA buffer (20 mM HEPES, pH 7.6, 350 mM NaCl, 1 mM MgCl_2_, 0.5 mM EDTA, 0.1 mM EGTA, 20% glycerol) supplemented with protease and phosphatase inhibitor tablets (Roche).

#### Reagents and Antibodies

The following reagents were used: zVAD-FMK (10 μM, Apex Bio), QVD (10 μM, Apex Bio), SM-164 (100 nM, gift from Shaomeng Wang), GSK’963 (referred as RIPK1i, 100 nM, GlaxoSmithKline), Nec-1 (10 μM Merck), Nec-1s (10 μM Merck), RO-3306 (9 μM, Merck), Thymidine (Sigma), BIM peptide (Kind gift of Tony Letai), FLAG-hTNF (10 ng/ml, Enzo), MG132 (1-20 μM, as indicated per cell line, see below, SIGMA), Recombinant Casp8 (1U Enzo). The following antibodies were used for western blotting: α-RIPK1 (1:1000, BD Biosciences), α-HA (1:1000, Roche), α-PLK1 (1:1000, Bethyl Laboratories), α-PLK1-pT210 (1:2000, AbCam), α-Cyclin B (1:1000, Cell Signaling), α-BUBR1 (1:1000, BD Bioscience), α-BUBR1-pT680 (1:1000, Kind gift of Geert J.P.L Kops), α-BUBR1-pT676 (1:1000, Kind gift of Erich A. Nigg), α-pH-H3 (1:2000, Millipore), α-Casp8 (for WB - post IP, 1:5000, MBL), α-Casp8 - for IP (7.5 μg/ml, C-20, Santa Cruz Biotechnology, α-FADD – for IP (7.5 μg/ml, Santa Cruz), α-FADD (1:1000 BD Biosciences), α-RIPK3 (1:1000 Proscience), α-RIPK3 (1:1000 Novus Biological) α-cFLIP (1:1000, Enzo), α-Myc (1:1000, clone 9E10,SIGMA), α-HSP90 (1:1000 Santa Cruz Biotechnology).

#### RNA Interference, Transfections

siRNAs were purchased from QIAGEN and Dharmacon. siRNA sequence information can be obtained upon request. All siRNA assays were performed using a total of 50 nM – 100 nM of siRNA. Upon multiple siRNAs combination, each siRNA was used at 25 nM and control siRNA was utilized to balance siRNA concentrations. All siRNA transfections were performed using DharmaFECT4 transfection reagent (GE Healthcare) and Opti-MEM (Life Technologies) according to manufacturer’s protocol. All siRNA transfections were performed using retro transfection and left for 40 hr from the time of transfection to induce knockdown. For overexpression studies, 5 × 10^5^ cells were plated, and the indicated constructs were transfected a day after according to the Genejuice transfection protocol (Merck Millipore). Cells were harvested 24 hr after transfection.

For the generation of CRISPR cells. Guide RNAs (gRNAs) were designed utilizing the website tool: www.crispor.tefor.net. Guide RNAs were cloned in pLC-mCherry vector, expressing Cas9. MDFs were seeded in a 6-well plate and transfected with 2 ug of CRISPR vector using Viromer yellow transfection reagent (Cambridge Bioscience) according to the manufacturer’s instructions. Three days later, cells were sorted using fluorescence-activated cell sorting (FACS), and single clones were isolated and screened for the deletion of RIPK1. Positive clones were characterized. Guide sequence utilized to delete RIPK1 is available upon request.

#### Cell synchronization

Cells were treated with 9 μM of CDK1i (RO-3306) for 20 hr. Inhibitor was washed out by 3 washes using full media, and cells were released in media containing DMSO (Ctrl) or media containing the indicated drugs. Asynchronized cells: cells not treated with CDK1i; G2 population: cells collected following 20 hr treatment with CDK1i; mitotic population: cells collected by shake off after a 2 hr release; non-M population: cells left at the bottom of the plate following mitotic shake off; G1 cells: cells collected 16 hr after release. For the scoring of chromosome misalignment, cells were released into media containing either DMSO or the indicated drugs for 30 minutes. After 30 min, cells were treated with 1 μM MG132 (HT1080), 10 μM MG132 (RPE-1 and primary MEFs) for 90 min. The addition of MG132 was added to assess the misalignments at metaphase plate (Janssen et al., 2009, Potapova et al., 2006) Cells were then fixed and processed as indicated above. Organoids were plated and treated with 4-OHT for 24 hr to allow deletion of *Ripk1*. 4-OHT was removed and cells were treated with CDK1i for 20 hr. Following 3x washes with media, organoids were released into media for 10 min. After 10 min, cells were treated with 20 μM MG132 for 90 min. Organoids were fixed for IF or lysed for western blotting. For the clonogenic assay, cells were synchronized with CDK1i for 20 hr, and released into media containing either DMSO or the indicated drugs. 30 min following release, cells were treated with MG132 for 90 minutes. Mitotic cells were collected by shake off, counted, and re-plated in media containing no drugs. For the clonogenic assay of non-M populations, cells were synchronized with CDK1i for 20 hr, then released into media for 16 hr. Subsequently, cells were treated for 2 hr with the indicated drugs. 30 min following treatment, media containing drugs was supplemented with MG132 for an additional 90 min. Cells were then trypsinised, counted, and re-plated for clonogenic growth in media containing no drugs.

#### Histology

E19.5 embryos were fixed in 10% neutral buffered formalin, paraffin embedded. E10.5 embryos were fixed in methanol, and agarose embedded. Embryos were then sectioned for routine histology staining (H&E). Slides were visualized and scored with a X100 objective. For immunofluorescent staining paraffin sections were dewaxed, subjected to heat-induced epitope retrieval with citrate buffer then blocked and permeabilised with 1% BSA and 0.3% Triton X-100 (for anti-CC3) or blocke in IFF buffer and permeabilised in 0.5% Triton X-100 (for anti-alpha Tubulin and DAPI). Images were taken using a DP72 microscope and cellSens Standard software (Olympus) for CC3 or with Zeiss LSM 710 for alpha Tubulin and DAPI.

#### Immunofluorescence

1x10^5^ cells were plated on 13 mm glass coverslips (VWR) and retro-transfected with siRNA or treated as indicated. Cells were then fixed in 4% PFA for 10 min. Following 10 min permeabilisation with 1x PBS, 0.5% Triton X-100, cells were blocked for 1 hr in 1x PBS, 5% BSA. Respective primary antibodies were diluted in 1x PBS, 1% BSA overnight at 4°C. These were: α-PLK1 (1:200 for PLA; 1:1000 for IF, for mouse PLA-Abcam 1:50), α-PLK1-pT210 (1:200 for DuoLink; 1:2000 for IF), α-BUBR1 (1:50 PLA, 1:1000 IF), α-BUBR1-pT680 (1:1000), α-BUBR1-pT676 (1:1000), α-FADD (1:50 for PLA), α- RIPK3 (1:50 PLA 1:1000 for IF), α-Casp8: C-20 (1:50 PLA, 1:1000 IF), for mouse PLA (Santa Cruz 1:50), α-cFLIP (1:50 PLA), α-RIPK1 (1:50 PLA, 1:1000 IF), α-α-Tubulin (Serotec 1:1000, for cells), α-α-Tubulin-488 (1:100, for organoids), DAPI (Invitrogen), α-human CREST serum (1:2000, kind gift from Stephan Geley), α-Phalloidin-633 (Invitrogen). Primary antibodies were washed 3x with 1x PBS, 0.1% Triton X-100 for 10 min. Secondary Alexa fluor-conjugated antibodies were diluted in 1x PBS, 1% BSA, and incubated for 1 hr at room temperature. These were: donkey α-rat-488 (1:1000, Invitrogen), donkey α-rat-633 (1:1000, Biotium), goat α-human-555 (1:1000, Invitrogen), goat α-rabbit-488 (1:1000, Invitrogen), donkey α-mouse-488 (1:1000, Biotium), goat α-rabbit-633 (1:1000, Invitrogen). Secondary antibodies were washed 3x with 1x PBS, 0.1% Triton X-100 for 10 min. DAPI was added to one of the three washes. Each slide was then washed with sterile water and mounted using Prolong Gold anti-fade fluorescent mounting media (Invitrogen). Organoids were fixed with 4% PFA for 1 hr at 37°C. These were then washed 3x in 1x PBS, and permeabilised for 1 hr using 1x PBS, 0.5% Triton X-100. Cells were blocked for 2 hr using IFF blocking buffer (1x PBS, 1% BSA, 2% FCS). Primary antibodies were diluted in IFF and left incubating overnight at 4°C, and then washed 3x with 1x PBS, 0.1% Triton X-100 for 30 min. Secondary fluorescent alexa fluor-conjugated antibodies were diluted in IFF buffer, left for 4 hr at room temperature, and washed 3x with 1x PBS, 0.1% Triton X-100 for 20 min. DAPI was added to each of these washes. The organoids chambers were further washed once in 1x PBS and once in H_2_O. Stained slides and organoids were visualized using the LSM710 Zeiss microscope, objective 40x or 63x Zeiss. Images were acquired by sequential scanning.

#### Live cell imaging

1x10^5^ HT1080 cells were seeded into 12-well plates (Porvoir), equilibrated into Leibovitz 15 (Life Technologies) medium supplemented with 10% FBS, 100 u/ml penicillin, 100 μg/ml streptomycin. Cells were incubated with 10 nM SIR-DNA and after treatments with the indicated drugs, cells were imaged every 6 min by advanced spinning disc confocal microscopy (Zeiss Axio Observer Z1, CSU-W1 T2 Spinning Disk Confocal, 50 μm Disk, Yokogama, Incubation from Oko laboratories with Temperature and Manual CO_2_ Control, Hamamatsu ORCA-Flash4.0 V2+ sCMOS Camera - USB3.0/30fps 1 with 82% peak QE, cooled to −10°C, 76. EC Plan-Neofluar 40x/0.75NA Objective, M27 1, working distance 0.71 mm, LaserStack 640 nm 100 mW, 692-40 filter). Only mitotic figures occurring within the first 5 hr of acquisition were taken into consideration for the analysis. Stacks and frames were acquired by SlideBook 6 Software System for Marianas. Movies were resolved by maximum projection and extracted as TIFF file and visualized by ImageJ. The mitotic duration was assessed by quantifying the time elapsing between nuclear envelope breakdown (NEBD) and anaphase onset.

#### Dynamic BH3 profiling (DBP)

Dynamic BH3 profiling was conducted as previously described (Ryan and Letai, 2013). Briefly, 0.2, 0.6, 2, and 6 μM of BIM BH3 peptide solution was prepared in DTEB (300 mM Trehalose, 10 mM HEPES-KOH [pH 7.7], 80 mM KCl, 1 mM EGTA, 1 mM EDTA, 0.1% BSA, 5 mM succinate). Cells were plated at a density of 5 × 10^5^ cells per well of a 6-well plate. Following synchronization, cells were collected and resuspended in DTEB at a density of 2.67x10^6^ (4x density). One volume of the cell suspension was added to one volume of a 4x dye solution (4 μM JC-1, 40 μg/ml oligomycin, 0.02% digitonin, 20 mM 2-mercaptoethanol in DTEB). The cell/dye solution was left at room temperature for 10 min to allow permeabilisation and dye equilibration. The cell/dye mix was then incubated with BIM BH3 peptides, and the mixture placed into the Infinite M200 pro TECAN plate reader. The plate was shaken for 15 s and individual wells were read at 590 nm over 3 hr, every 5 min at 30°C. The obtained values were plotted as Δ priming. The difference in priming was determined by calculating the area under curve obtained following treatment with DMSO (control) and FCCP (control) minus the area under the curve obtained following treatment with the individual peptide concentrations and FCCP. These differences where then plotted as ΔΨn relative fluorescent unit (RFU) measured at 590 nm and normalized to asynchronously growing cells.

#### DNA Plasmids

PLK1 cDNA constructs were purchased from ADDgene. All constructs used for transient transfection experiments were cloned into the pcDNA3 expression vector (Invitrogen) and sequence verified.

#### Caspase activity assays (DEVDase)

1x10^4^ cells were plated in 96-well plates and retro siRNA transfection was performed for 40 hr. For the caspase activity assays during prolonged mitosis, cells were plated in 96-well plates and treated as indicated for the indicated time points. After treatment, medium was removed and 20 μL of DISC lysis buffer (20 mM Tris-HCL pH 7.5, 150 mM NaCl, 2 mM EDTA, 1% Triton X-100, 10% glycerol) was added to each well. Pellets were washed in 1x PBS and lysed in DISC lysis buffer. Plates were placed at −80°C to aid cell lysis. Plates were thawed at room temperature for 15 min, after which 180 μL DEVDase assay mix (20 μM Ac-DEVD-AMC (Sigma), 1 mM DTT, 50 mM Tris pH 7.5, 150 mM NaCl, 0.1% Triton X-100, and 5% glycerol) was added to each well (NB: cell lysates were not cleared). The plates were wrapped in foil and the reaction was incubated at room temperature for up to 24 hr. DEVDase activity was read at 380 nM excitation/460 nM emission. For Mitotic population, cells were collected by mitotic shake off, then centrifuged for 5 min at 2000 rpm. These were then lysed in DISC lysis buffer. All the other samples were trypsinised and cells were collected by centrifugation for 5 min at 2000 rpm as for the mitotic population. Following addition of lysis buffer, samples were incubated at −80°C to aid cell lysis. 50 μL of each samples was added to a 24-well plate and 450 μL of DEVEase assay mix was added to each well. Caspase activity was then measured as indicated above. The remaining of these samples was boiled and protein amounts were quantified. These were then utilized to normalize the individual caspase activity of each sample.

#### *In vitro* caspase cleavage assay

Recombinant Casp8 (2 U) and/or PLK1 (1 μg) were incubated in caspase cleavage buffer (50 mM HEPES, pH 7.2, 50 mM sodium chloride, 0.1% CHAPS, 10 mM EDTA, 5% Glycerol, 10 mM DTT) for 1 and 2 hr at 37°C. Samples were then diluted in 2X sample buffer and analyzed by western blotting.

#### Cell Death assay (FACS)

Cell mixtures were incubated with medium containing 1 μg/ml propidum iodide and analyzed by FACS using a plate reader. Data shown are from 5000-10000 cells per condition. For cell viability of parental and Bcl-2 overexpressing HT1080 cells were assessed using a high-content imaging system (ImageXpress Micro-XL, Molecular Devices). Cells were plated in 96-well thin bottom plates in triplicate wells, and treated as indicated. A 20x long distance objective was used to image 16 sites in each well. Values were averaged for the 16 sites for each well, and shown are the mean ± SEM of 3 wells for each condition. Before imaging cells were loaded with 40 nM SYTOX Green nucleic acid stain (ThermoFisher Scientific), 1 μg/mL Hoechst 33342 (Sigma) for 30 min. The dyes were present during imaging. Images were analyzed using the Multiwavelength Cell Scoring module of the MetaXpress software (Molecular Devices). Cells were identified based on Hoechst staining, then nuclear SYTOX Green intensity was scored to identify cells with permeabilised plasma-membrane (SYTOX positive cells). Scoring was based on the size of area above threshold, determined by intact, non-treated cells.

#### Clonogenic Assays

1000 cells were left for approximately 10 days in medium. Cells were fixed in 4% PFA/PBS for 10 min and stained with crystal violet/PBS for 20 min. Colonies were counted and recorded using ImageJ.

#### Immuno-Precipitation Assays

Cells were lysed in DISC lysis buffer (20 mM Tris-HCL pH 7.5, 150 mM NaCl, 2 mM EDTA, 1% Triton X-100, 10% glycerol) supplemented with protease and phosphatases inhibitors. Cell lysates were left in the −80°C to aid cell lysis for 20 min, then rotated at 4°C until thawed. Cell lysates were collected by centrifugation at 4°C at 14000 rpm for 15 min. 20 μL of G agarose (SIGMA); 7.5 μL Casp8 antibody/mg protein lysate (Santa Cruz) or 5 μL of Myc antibody/mg of protein lysate, were rotated with cleared protein lysates overnight at 4°C. 5x washes in wash buffer (1x PBS, 1% Triton X-100, 1 mM EDTA, 5% glycerol) were performed, and samples eluted by boiling for 5 min in 60 μL of 2x SDS loading dye.

#### Proximity Ligation Assay

1x10^5^ cells were plated on a 13 mm glass coverslip (VWR) and retro-transfected with siRNA as indicated. Cells were then synchronized as described above and fixed in 4% PFA for 10 min. Following 10 min permeabilisation with 1x PBS, 0.5% Triton X-100, cells were blocked for 1 hr in 1x PBS, 5% BSA. Respective pair of primary antibodies were then added as indicated. Proximity ligation was performed according to the manufacturer’s instructions using the Duolink Detection Kit (Cambridge BioScience Ltd, Cambridge UK). Ligation and amplification reactions were carried out according to manufacturer’s instructions. Cy3 signal amplification was utilized for the assay. Following PLA, cells were immune-blocked with α-rat α-tubulin (Serotec) diluted in PBS 1% BSA for 1 hr. Following three washes with 1x PBS, 0.1% Triton X-100, slides were incubated with donkey α-rat alexa fluor-633 diluted in 1x PBS, 1% BSA for 1 hr. Secondary antibody was washed three time with 1x PBS, 0.1% Triton X-100, and stained with DAPI for 10 min. Each slide was then washed with sterile water and mounted using Prolong Gold anti-fade fluorescent mounting media (Invitrogen) and left to air dry overnight. Cells were examined with a confocal microscope (objective 40x, Zeiss LSM 710).

#### Mass Spectrometry

Prior to mass spectrometry analysis of RIPK1 interactors, eluted protein complexes were digested with Trypsin and peptides were purified using C18 Microspin columns (Harvard Apparatus) according to the manufactures instruction. LC-MS/MS analysis was performed on a dual pressure LTQ-Orbitrap mass spectrometer (Thermo Scientific), which was connected to an electrospray ion source (Thermo Scientific). Peptide separation was carried out using an easy nano-LC systems (Proxeon Biosystems) equipped with an RP-HPLC column packed with C18 resin (Magic C18 AQ 3 μm; Michrom BioResources). A 0.3 μl/min linear gradient from 96% solvent A (0.15% formic acid, 2% acetonitrile) and 4% solvent B (98% acetonitrile, 0.15% formic acid) to 40% solvent B over 40 min. The data acquisition mode was set to obtain one high-resolution MS scan in the FT part of the mass spectrometer at a resolution of 60,000 FWHM followed by MS/MS scans in the linear ion trap of the 20 most intense ions. Raw files were converted to the mzXML format, and searched against the human swissprot protein database. Further data processing including SAINT was carried out as described previously (Choi et al., 2011).

#### Cell cycle profile

5x10^5^ cells where plates in a six well plate. Cells were synchronized as described above. Following synchronization and release in media containing the indicated drugs, cells were fixed in 70% EtOH for 24 hr, and stained with rabbit alexa fluor 488 (pH-H3) and propidium iodide (PI). 5000 Cells were then analyzed via FACS, and percentage of different cell cycle stages were obtained.

### Quantification and Statistical Analysis

#### Experimental procedure

Each experiment was conducted at least three times to generate three biological repeats. For western blots, immunoprecipitations and IF experiment the most representative experiment is shown. Bar graphs for quantifications show the average of three independent experiments the SE and P value generated following two-way ANOVA multiple comparison test. No adjustments for multiple comparison was utilized. Number (n) of cell counted per experiment is noted for each experiment either in the legend or on the figure.

#### Statistics

Data shown represent the mean ± SD or SEM, as indicated in the figure legends. Two-way ANOVA multiple comparison analysis was performed for all the data unless otherwise indicated using Prism6. ^∗^p < 0.05 ^∗∗^p < 0.01 ^∗∗∗^p < 0.001.

### Data and Software Availability

Raw data have been deposited to Mendeley Data and are available at http://doi:10.17632/x8tvmp6rfb.1

## Supplemental Information

Document S1. Figures S1–S7

Document S2. Article plus Supplemental Information
