# Supplementary material for: RIPK1 and Caspase-8 Ensure Chromosome Stability Independently of Their Role in Cell Death and Inflammation
Source: Mol Cell. 2019 Feb 7;73(3):413–428.e7. doi: 10.1016/j.molcel.2018.11.010 (PMC6375735; doi:10.1016/j.molcel.2018.11.010)
Supplement: Document S1. Figures S1–S7 [file mmc1.pdf]

Molecular Cell, Volume *73*

Supplemental Information

RIPK1 and Caspase-8 Ensure Chromosome Stability Independently of Their Role in Cell Death and Inflammation

Gianmaria Liccardi, Laura Ramos Garcia, Tencho Tenev, Alessandro Annibaldi, Arnaud J. Legrand, David Robertson, Rebecca Feltham, Holly Anderton, Maurice Darding, Nieves Peltzer, Marius Dannappel, Hannah Schünke, Luca L. Fava, Manuel D. Haschka, Timo Glatter, Alexey Nesvizhskii, Alexander Schmidt, Philip A. Harris, John Bertin, Peter J. Gough, Andreas Villunger, John Silke, Manolis Pasparakis, Katiuscia Bianchi, and Pascal Meier

Supplementary Information

Supplementary Figures:

Figure S1

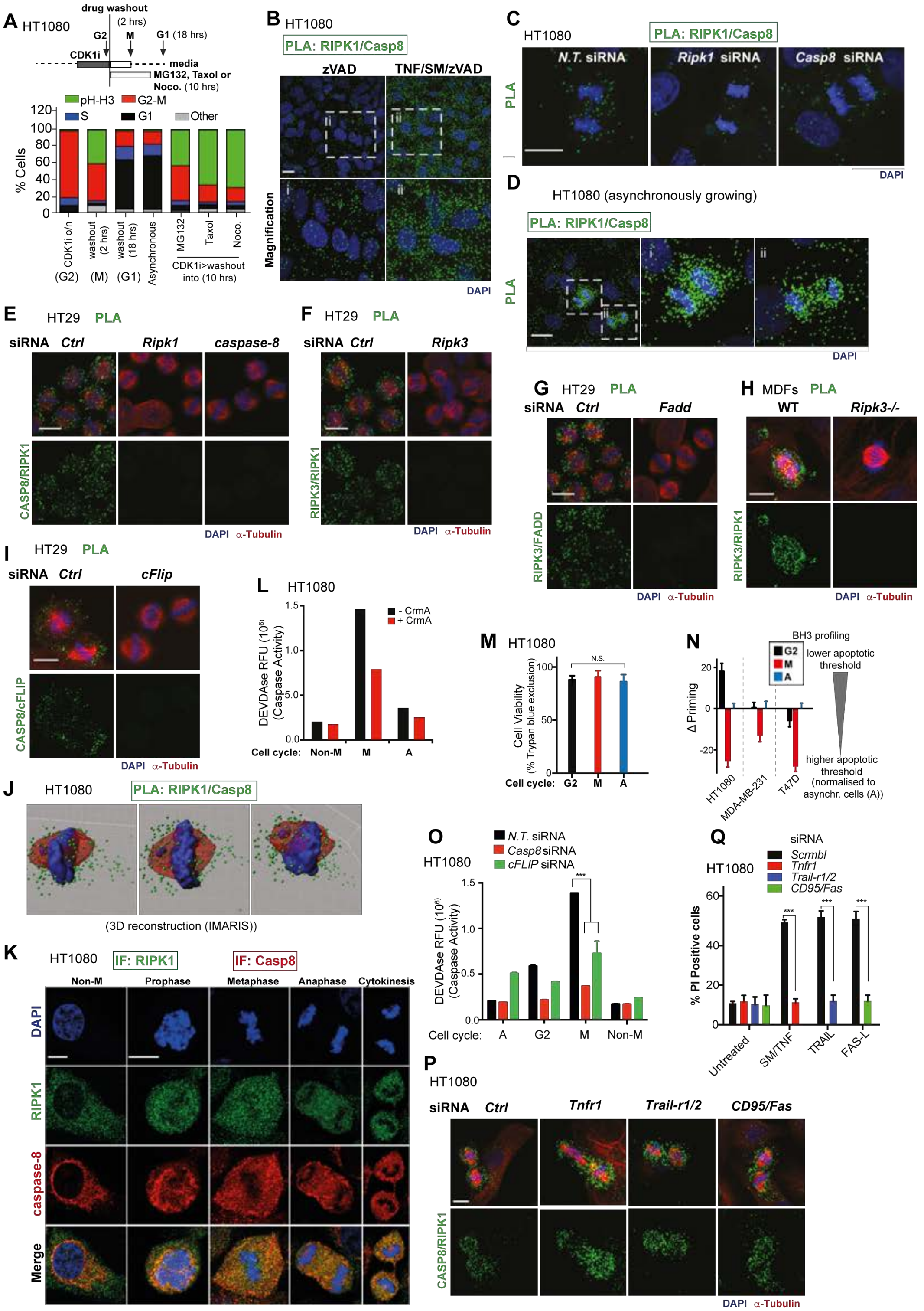

Figure S1 related to Figure 1 | The ripoptosome forms during normal mitosis.

(A) FACS analysis of the cell cycle profile of CDK1i-synchronised and released HT1080 cells. Cells were stained with PI for DNA content and with pH-H3 (alexa fluor 488) to detect mitotic cells. Graphs indicate the synchronisation procedure and the time of cell collections for FACS analysis.

(B-D) *In situ* PLA detection of RIPK1 and Casp8 in HT1080 cells treated with the indicated conditions. Green dots indicate proximity signals between RIPK1 and Casp8 in asynchronously growing cells treated with zVAD or TNF/SM/zVAD (B), or CDK1i-synchronised and released cells upon the indicated knockdown (C), or asynchronously growing cells (D). (Scale bars: 10 μm).

(E-I) *In situ* PLA detection in HT29 (E-G, I) and Mouse dermal Fibroblasts (MDFs) (H) or CDK1i-synchronised and released cells upon the indicated knockdown. Green dots indicate proximity signals between RIPK1 and Casp8 (E) RIPK1 and human-specific RIPK3 (F) RIPK1 and mouse-specific RIPK3 (H) human-specific RIPK3 and FADD (G) and Casp8 and c-FLIP (I) (Scale bars: 10 μm).

(J) 3D-reconstruction (IMARIS) of ripoptosome complexes visualised by PLA.

(K) Immunofluorescence analysis using anti-RIPK1 and anti-Casp8 antibodies. HT1080 cells were synchronised with CDK1i and released into media. Scale bars: 10 μm.

(L) DEVDase caspase activity analysis using synchronised and released HT1080 cells in the presence or absence of CrmA. For this experiment, we used HT1080 that carry an inducible construct of CrmA (Tenev et al., 2011). The graph shows a representative experiment of three independent experiments.

(M) Viability assay of CDK1i-synchronised and released HT1080 cells. The graph shows the mean ±SE of three independent experiments.

(N) BH3 profile of CDK1i-synchronised and released HT1080 cells. Graphs show the mean ±SD of three experiments. The differences in priming was plotted as $\Delta\Psi$n relative fluorescent unit (RFU) and normalized to asynchronously growing cells.

(O) DEVDase caspase activity analysis using CDK1i-synchronised and released HT1080 cells treated with the indicated conditions.

(P) *In situ* PLA detection in CDK1i-synchronised and released HT1080 cells upon the indicated knockdown. Green dots indicate proximity signals between RIPK1 and Casp8 (Scale bars: 10 μm).

(Q) Functional validation of the knockdown efficiencies. Cell death assay of HT1080 transfected with the indicated siRNA oligos following indicated treatment (24 hrs). Graphs show the mean ±SD of three independent experiments. Two-way Anova multiple comparison analysis with $^{***}P<0.001$

Figure S2

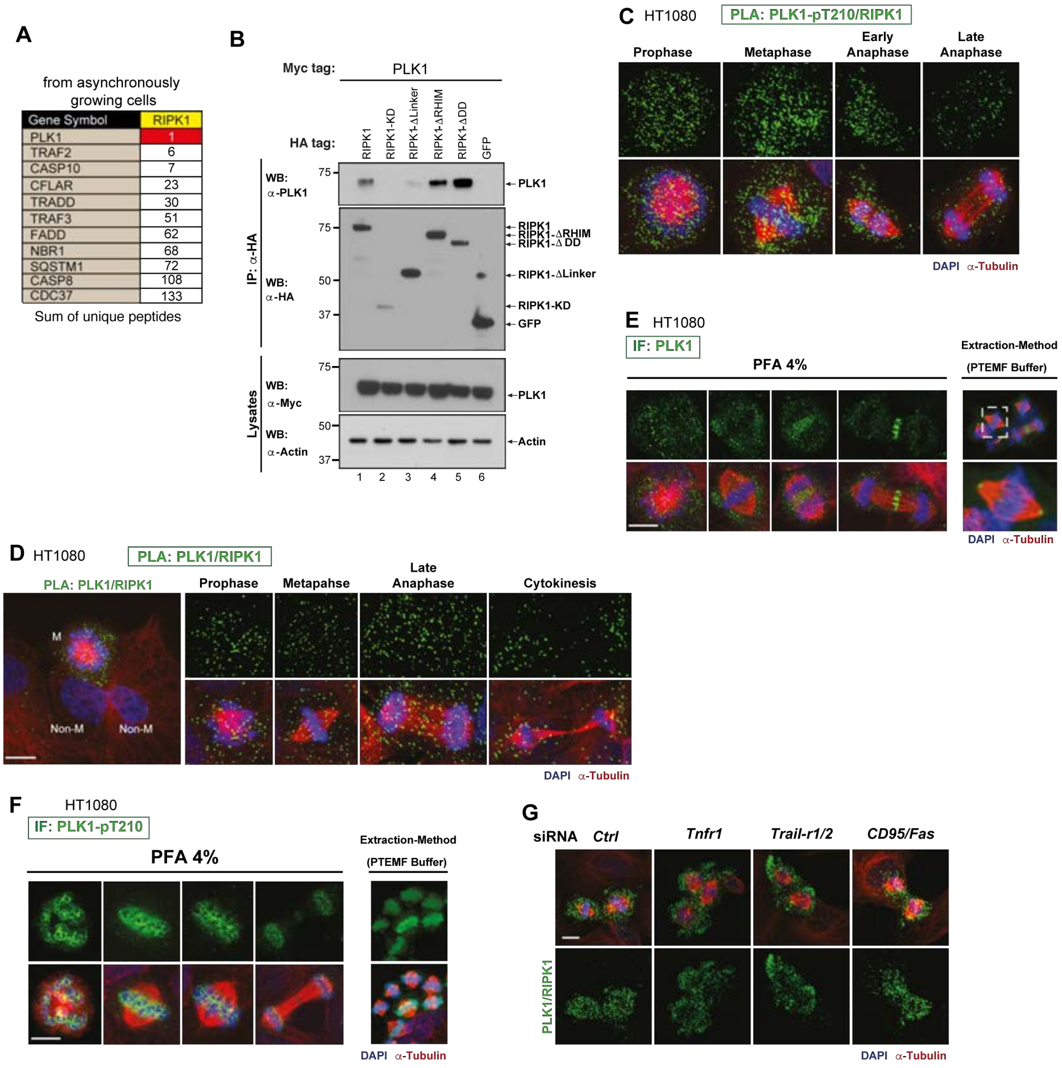

Figure S2 related to Figure 2 | RIPK1 interacts with PLK1.

(A) Schematic representation of RIPK1-bound target proteins that were identified by mass spectrometry. The table specifies the sum of the unique peptides of the target protein identified.

(B) The indicated constructs were co-expressed in HEK293T cells. HA-immunoprecipitation was performed and PLK1 interaction was assessed via Western blot.

(C-D) *In situ* PLA detection in CDK1i-synchronised and released HT1080 cells. Green dots indicate proximity signals between RIPK1 and pPLK1-T210 (C) and RIPK1 and PLK1 (D) (Scale bars: 10 μm).

(E-F) Immunofluorescence analysis using anti-PLK1 (E) and anti-PLK1-pT210 (F) antibodies using different fixation conditions. HT1080 cells were synchronised with CDK1i and released into media. Scale bars: 10 μm

(G) *In situ* PLA detection in CDK1i-synchronised and released HT1080 cells upon the indicated knockdown. Green dots indicate proximity signals between RIPK1 and PLK1 (Scale bars: 10 μm).

Figure S3

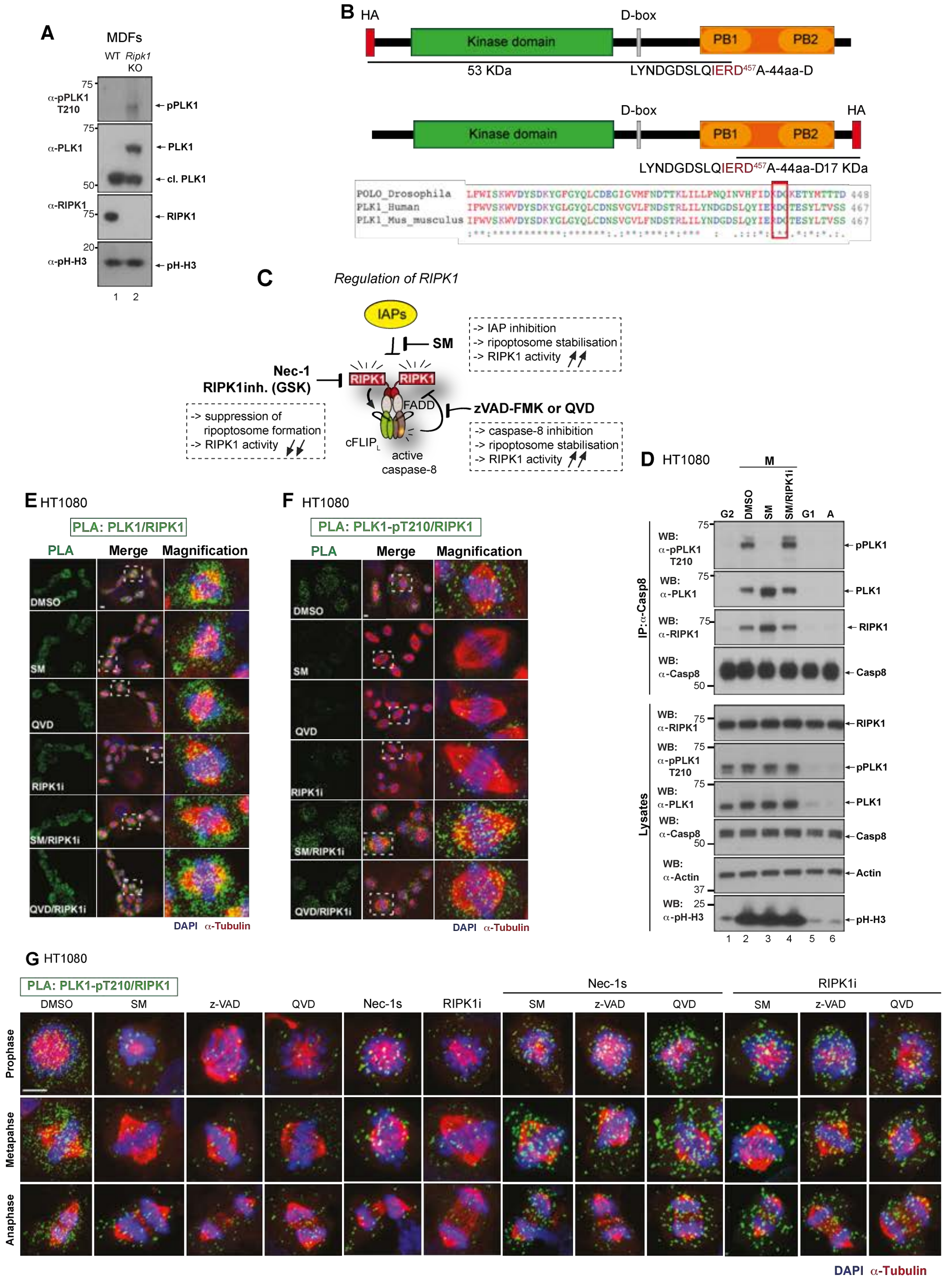
A
MDFs
WT Ripk1 KO
α-pPLK1 T210
α-PLK1
α-RIPK1
α-pH-H3
pPLK1
PLK1
cl. PLK1
RIPK1
pH-H3
B HA
D-box
Kinase domain
PB1
PB2
53 KDa
LYNDGDSLQIERD457A-44aa-D
HA
LYNDGDSLQIERD457A-44aa-D17 KDa
C
Regulation of RIPK1
IAPs
SM
-> IAP inhibition
-> ripoptosome stabilisation
-> RIPK1 activity
Nec-1
RIPK1inh. (GSK)
RIPK1
FADD
zVAD-FMK or QVD
-> suppression of ripoptosome formation
-> RIPK1 activity
cFLIP
active caspase-8
-> caspase-8 inhibition
-> ripoptosome stabilisation
-> RIPK1 activity
D HT1080
E HT1080
PLA: PLK1/RIPK1
PLA
Merge
Magnification
F HT1080
PLA: PLK1-pT210/RIPK1
DMSO
SM
QVD
RIPK1i
SM/RIPK1i
QVD/RIPK1i
DAPI α-Tubulin
G2
M
G1
A
IP:α-Casp8
Lysates
WB: α-pPLK1 T210
WB: α-PLK1
WB: α-RIPK1
WB: α-Casp8
WB: α-Actin
WB: α-pH-H3
Casp8
Actin
G HT1080
PLA: PLK1-pT210/RIPK1
Nec-1s
RIPK1i
z-VAD
Prophase
Metapahse
Anaphase

Figure S3 related to Figure 3 | RIPK1 negatively regulates PLK1.

(A) Immunoblots of mouse dermal fibroblasts where *Ripk1* was ablated by CRISPR/Cas9. Cells were synchronized with CDK1i and released into media for 2 hours and then lysed and analysed by immunoblotting with the indicated antibodies

(B) Schematic representation of the PLK1 constructs utilised in the *in vitro* cleavage assay. Alignment of Drosophila, Human and Mouse *Plk1* sequence highlighting the identified cleavage site.

(C) Schematic representation of RIPK1 regulation, and the drugs utilised to modulate RIPK1's kinase and scaffolding function.

(D) Synchronised HT1080 cells were released in media containing the indicated drugs. Lysates were immunoprecipitated with anti-Casp8 antibody. Immunoblot analysis using the indicated antibodies is shown.

(E-G) *In situ* PLA detection of PLK1/RIPK1 (E) or PLK1-pT210/RIPK1 (F-G) in CDK1i-synchronised and released HT1080 cells treated with the indicated agents. Green dots indicate PLA speckles. Scale bars: 10 μm.

Figure S4

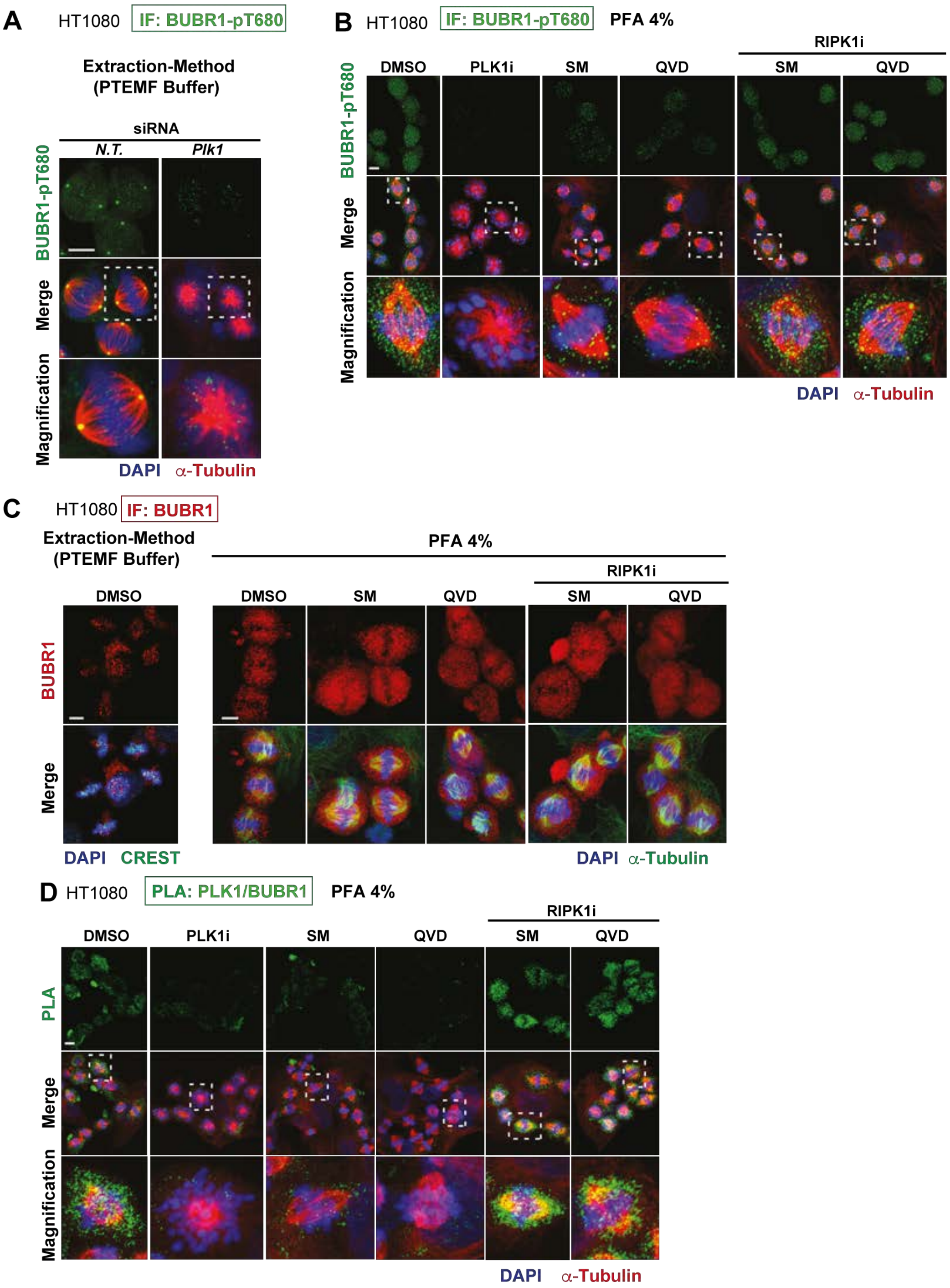

Figure S4 related to Figure 4 | RIPK1 negatively regulates PLK1-mediated phosphorylation of BUBR1.

(A-C) Immunofluorescence analysis using CREST serum, anti-BUBR1-pT680 and anti-BUBR1 antibodies using different fixation conditions. HT1080 cells were synchronised with CDK1i and released into media. Scale bars: 10 μm.

(D) *In situ* PLA detection of PLK1 and BUBR1 in HT1080 cells, treated with the indicated agents. Green dots indicate proximity signals between PLK1 and BUBR1. Scale bars: 10 μm.

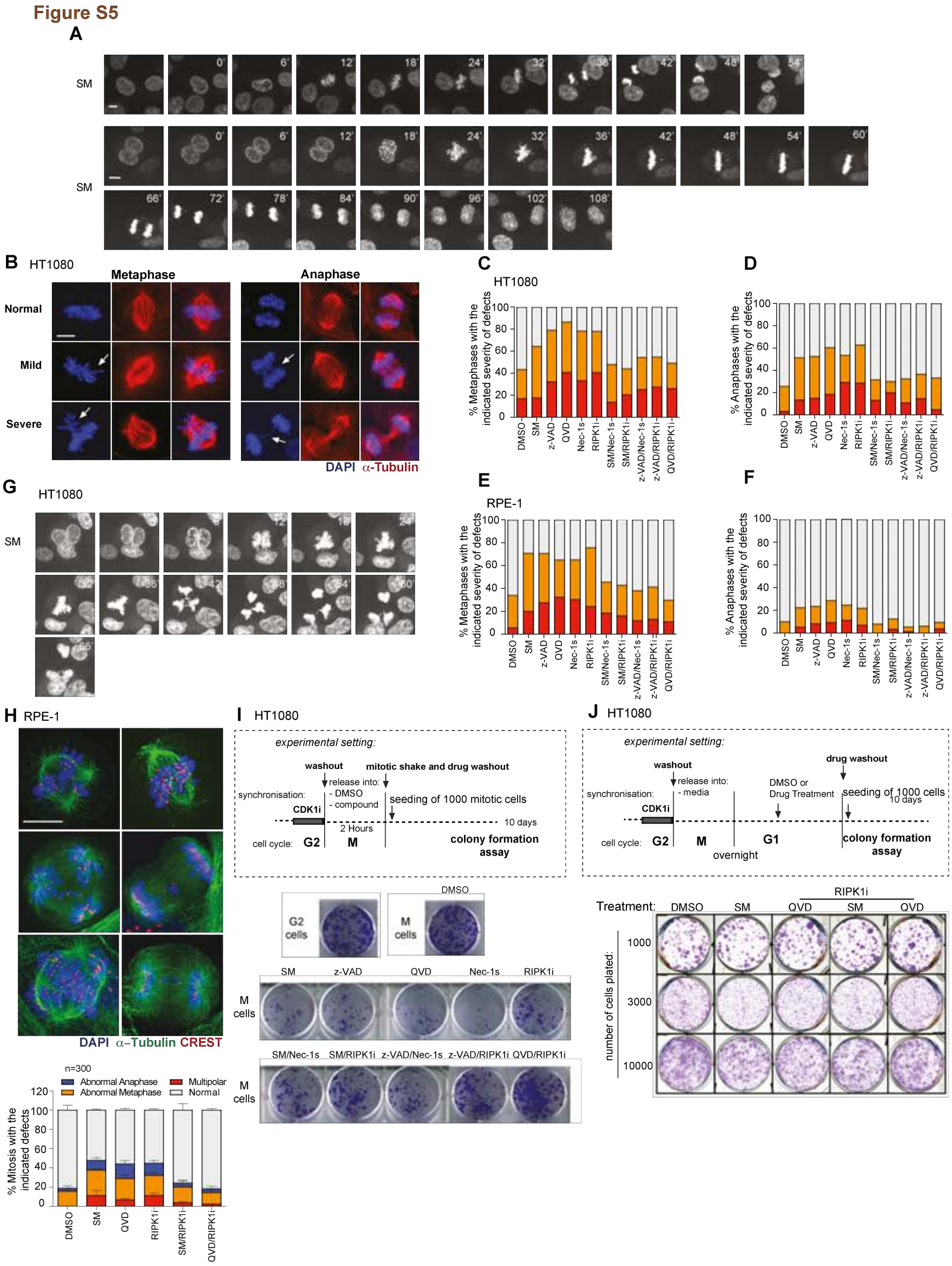

Figure S5 related to Figure 5 | Hyper-activation of RIPK1 induces chromosome misalignment.

(A) Example of mitotic cells visualised during advance spinning confocal time lapse. Each frame was acquired every 6 mins.

(B) Examples of chromosome alignment defects to illustrate the scoring system. Scale bars: 10 μm.

(C-F) CDK1i-synchronised HT1080 (B and C) and RPE-1 (D and E) cells were released into media containing the indicated agents. For the analysis in metaphases, cells were released for 30 mins after which MG132 was added for 90 mins to arrest cells in metaphase. Anaphases were scored after 2 hrs release. 100 mitosis were counted for each condition per experiment.

(G) Example of mitotic cells visualised during advance spinning confocal time lapse. Each frame was acquired every 6 mins.

(H) CDK1i-synchronised RPE-1 cells were released into media containing the indicated agents. For the analysis in metaphases cells were released for 30 mins after which MG132 was added for 90 mins to arrest cells in metaphase. Anaphases and Multipolar mitosis were scored after 2 hrs release. Figure shows representative images of the collected phenotypes. Graphs show the mean ±SE of two independent experiments. Scale bars: 10 μm.

(I) Synchronised HT1080 cells were released into media containing the indicated drugs for 30 minutes and then treatment was supplemented with MG132 for 90 minutes. Mitotic cells were collected by shake off and 1000 cells were plated for clonogenic assay

(J) CDK1i-synchronised HT1080 cells were release into media for 20 hrs and then released in media for 30 mins, after which cells were treated with MG132 for 90 minutes. 16 hrs following release cells were treated with the indicated drugs for 2 hrs. Cells were collected by trypsinisation, washed and the indicated number of cells were plated for clonogenic assay.

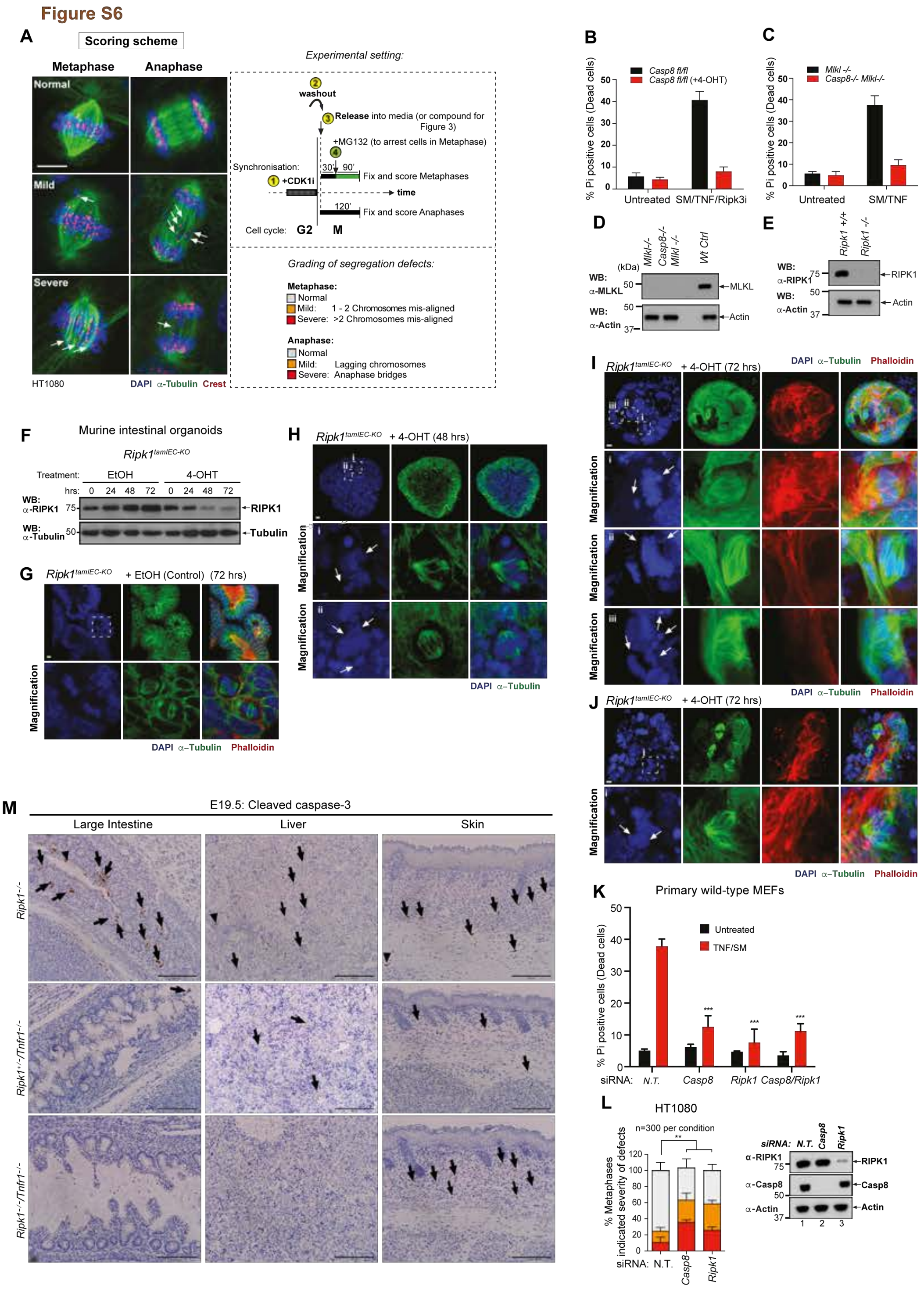

Figure S6 related to Figure 6 | Deficiency of RIPK1 or Casp8 results in chromosome alignment defects

(A) Grading of segregation defects. The scheme indicates the experimental procedure as detailed in material and methods.

(B-C) Functional validation of primary MEFs following treatment with SM/TNF (24 hrs). Genetic deletion is indicated. Graphs show the mean ±SD of three independent experiments. Two-way Anova multiple comparison analysis with ****P*<0.001.

(D-E) Western blot validation of primary MEFs with the indicated genetic deletions.

(F) RIPK1 immunoblots of $Ripk1^{tamIEC\text{-}KO}$-derived organoids treated with vehicle control (EtOH) or 4-OHT for 20 hrs. Organoid cultures were analysed at the indicated time points.

(G-J) Images depict $Ripk1^{tamIEC\text{-}KO}$-derived organoids treated with EtOH (G) or 4-OHT (H-J). Arrows indicate chromosomal misalignment. i,ii,iii and square indicate abnormal mitosis. Images show chromosomal abnormalities before and after loss of intestinal architecture. Scale bars: 10 μm.

(K) Functional validation of the knockdown efficiencies. Cell death assay of primary MEFs transfected with the indicated siRNA oligos following treatment with SM/TNF (24 hrs). Graphs show the mean ±SD of three independent experiments. Two-way Anova multiple comparison analysis with ****P*<0.001

(L) Chromosome alignment defects of synchronised and released HT1080 cells in which the indicated genes were knocked-down by RNAi. Graphs show the mean ±SE of three independent experiments. Two-way Anova multiple comparison analysis with ****P*<0.001. Total amount of abnormalities was considered in determining statistical significance.

Levels of knockdown were validated by Western blot analysis.

(M) Tissue sections of the indicated genotypes stained with cleaved caspase-3 (CC3) (brown) and hematoxylin (blue). Brown cells are positive for CC3. Black arrows indicate CC3 positive cells.

Figure S7

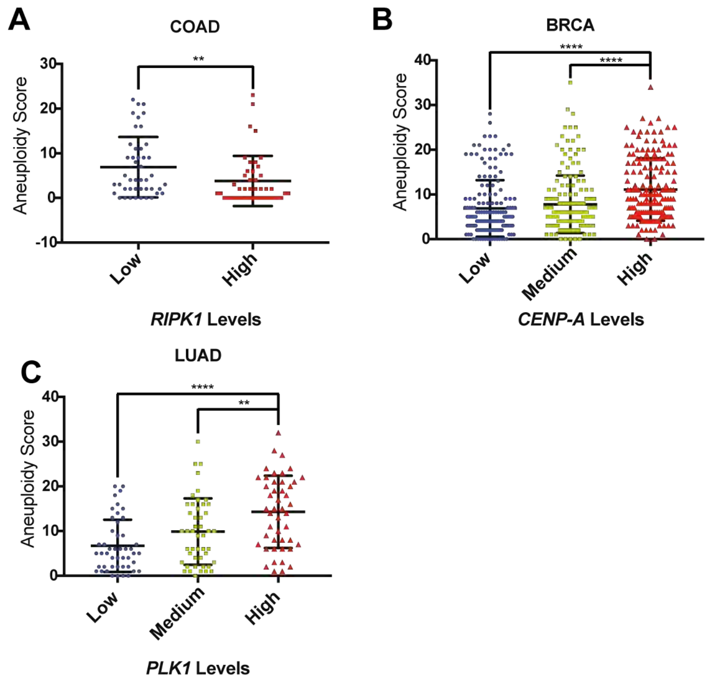

Figure S7 related to Figure 7 | *RIPK1* mRNA levels correlate with aneuploidy in Human cancers

(A-C) Bioinformatics analyses of aneuploidy scores in association with *RIPK1* mRNA expression (A), *CENP-A* mRNA expression (B) and *PLK1* mRNA expression (C) in colorectal (A), breast (B) and lung (C) cancer patients. COAD: Colorectal Adenocarcinoma; BRCA: Breast Cancer; LUAD: Lung Adenocarcinoma. **: p<0.01; ****p<0.0001. (A) *n*: 89; (B) *n*: 516; (C) *n*: 140.

Supplementary items. Supplementary movies 1-3 related to Figure 5 | Modulation of RIPK1 induces Chromosomal instability.

Asynchronised HT1080 cells were pre-incubated for two hours with 10 nM SIR-DNA and then treated with the indicated compounds: DMSO, SM, SM/RIPK1i. Live cell imaging was recorded by advance spinning confocal time lapse filming. Frames were acquired every 6 mins for 10 hours. Only the first 5 hours (90 frames) were taken in consideration. Movies should be opened via ImageJ and colour balance should be adjusted according to the user preferences.
